# WIPF1 antagonizes the tumor suppressive effect of miR-141/200c and is associated with poor survival in patients with PDAC

**DOI:** 10.1186/s13046-018-0848-6

**Published:** 2018-07-24

**Authors:** Yu Pan, Fengchun Lu, Ping Xiong, Maoen Pan, Zheyang Zhang, Xianchao Lin, Minggui Pan, Heguang Huang

**Affiliations:** 1 Department of General Surgery, Fujian Medical University Union Hospital, No.29 Xinquan Road, Fuzhou, 350001 People’s Republic of China; 2 Department of obstetrics and gynecology, Fuzhou General Hospital, 156 North Xi-er Huan Road, Fuzhou, 350001 China; 3 College of Bioinformatics Science and Technology, Harbin Medical University, Harbin, 150086 China; 4 Department of Oncology and Hematology and Division of Research, Kaiser Permanente, Santa Clara, CA 95051 USA

**Keywords:** miR-141, miR-200c, WIPF1, YAP/TAZ, Pancreatic cancer

## Abstract

**Background:**

Aberrant expression of Wiskott–Aldrich syndrome protein interacting protein family member 1 (WIPF1) contributes to the invasion and metastasis of several malignancies. However, the role of WIPF1 in human pancreatic ductal adenocarcinoma (PDAC) remains poorly understood.

**Methods:**

Human pancreatic cancer samples from PDAC patients were collected for methylation analysis. Bioinformatic prediction program and luciferase reporter assay were used to identify microRNAs regulating WIPF1 expression. The association between WIPF1 expression and the overall survival (OS) of patients with PDAC was evaluated by using The Cancer Genome Atlas (TCGA) database. The roles of miR-141/200c and WIPF1 on the invasion and metastasis of PDAC cells were investigated both in vitro and in vivo.

**Results:**

We found that compared to the surrounding non-cancerous tissues, there was significantly increased methylation of miR-200c and miR-141 in human PDAC tissues that resulted in their silencing, whereas the members of the other cluster of miR-200 family including miR-200a, miR-200b and miR-429 were hypomethylated. Our data show that forced expression of miR-141 or miR-200c suppressed invasion and metastasis of PDAC cells both in vitro and in xenograft and identified WIPF1 as a direct target of miR-141 and miR-200c. Both miR-141 and miR-200c inhibit WIPF1 by directly interacting with its 3′-untranslated region. Remarkably, silencing of WIPF1 blocked PDAC growth and metastasis both in vitro and in vivo, whereas forced WIPF1 overexpression antagonized the tumor suppressive effect of miR-141/200c. Additionally, by using TCGA database we showed that high expression of WIPF1 correlated with poor survival in patients with PDAC. Moreover, we show that miR-141 and miR-200c blocked YAP/TAZ expression by suppressing WIPF1.

**Conclusions:**

We have identified WIPF1 as an oncoprotein in PDAC and a direct target of miR-141/miR-200c. We have also defined the miR-141/200c-WIPF1-YAP/TAZ as a novel signaling pathway that is involved in the regulation of the invasion and metastasis of human PDAC cells.

**Electronic supplementary material:**

The online version of this article (10.1186/s13046-018-0848-6) contains supplementary material, which is available to authorized users.

## Background

Pancreatic ductal adenocarcinoma (PDAC) is a highly aggressive malignancy and one of the leading causes of cancer death worldwide with very poor 5-year overall survival (OS) [1, 2]. For early stage patients, surgical resection remains the mainstay of treatment, followed by post-operative chemotherapy with or without radiation. Most patients are unfortunately diagnosed with advanced stage, and for patients with early stage disease, relapse is extremely common [3–5]. Therefore, it is imperative to investigate for potential therapeutic strategies by understanding the mechanism of invasion and metastasis of PDAC.

Several microRNAs have been identified to be involved in the oncogenic process, drug resistance, and metastasis of PDAC [6]. The CpG methylation of microRNA promoter region has been found to be a major mechanism in regulating their expression [7]. The microRNA miR-200 gene family (including miR-200a, miR-200b, miR-200c, miR-141 and miR-429) which is clustered in two separate chromosomal locations: miR-200a/200b/429 on chromosome 1 and miR-141/200c on chromosome 12, has been characterized with high methylation and low expression in a variety of tumor cells. Downregulation of miR-141, miR-200a, miR-200b and miR-200c have been shown to promote cancer cell proliferation and metastasis in prostate cancer [8], gastric cancer [9], hepatocellular carcinoma [10], head and neck squamous cell carcinoma [11]. In addition, miR-200c/429 was shown to suppress tumorignecity and invasion of breast cancer stem cells [12, 13]. A study showed that miR-200a and miR-200b were hypomethylated in pancreatic cancer, distinct from the other findings in other malignancies suggesting that miR-200a/b may promote cell proliferation in pancreatic cancer [14]. The methylation status of other miR-200 members in PDAC has not been reported yet. Furthermore, most studies exploring the functional role and target genes of miR-200 in PDAC have depended on cell culture experiments with little in vivo characterizations [15–18].

The gene encoded by Wiskott–Aldrich syndrome protein (WASP) interacting protein family member 1 (WIPF1) participates in actin cytoskeleton organization and polymerization that are associated with cell proliferation and invasion [19–21]. WIPF1 binds to a region of Wiskott-Aldrich syndrome protein (WASP) that is frequently mutated in Wiskott-Aldrich syndrome (WAS) [22, 23]. WAS is associated with high frequency of malignancy especially lymphoma [24]. WIPF1 was found to be a oncogene in breast cancer, glioma and colorectal cancer [25]. However, the role of WIPF1 in PDAC is unclear.

YAP/TAZ is a key component of HIPPO signaling pathway that is consisted of a kinase cascade controlling organ size by regulating cell proliferation and differentiation [26]. While many components of HIPPO pathway are tumor suppressors, YAP/TAZ functions as oncogene by suppressing the contact inhibition, conferring the stemness and stimulating cell growth and metastasis of malignant cells [27–30]. Like WIPF1, YAP/TAZ is overexpressed in several malignancies including PDAC and has also been shown to regulate cytoskeleton organization of cells and cell adhesion [28, 29].

The aim of this study was to investigate the mechanism by which miR-200 family members regulate the oncologic behaviors of PDAC. We show that miR-141/200c is epigenetically silenced in PDAC both in vitro and in vivo. Furthermore, we identified WIPF1 as a direct target of miR-141/200c and demonstrated that miR-141/200c interacts with the 3′-untranslated region of WIPF1 to inhibit WIPF1-YAP/TAZ pathway in PDAC, finally suppressing PDAC growth and metastasis.

## Methods

### Patients and tissue samples

This study was approved by the Committee for the Ethical Review of Research, Fujian Medical University Union Hospital (No. 2016-ZQN-34). Human pancreatic cancer samples were collected from patients undergoing surgery at Fujian Medical University Union Hospital, Fuzhou, China, from March 2016 to July 2017. Informed consent was obtained before sample collection. All patients received curative surgery and had histologically confirmed PDAC. None of the patients received neoadjuvant radiation or chemotherapy. The stage of each patient was assessed based on the American Joint Committee on Cancer version 7 (AJCC 7). The tissue samples were placed in liquid nitrogen or RNAlater immediately after dissection and stored at −80C° until DNA and RNA extractions were performed.

### Cell culture and drug treatment

The human pancreatic duct epithelial cells (HPDE) and pancreatic cancer cell lines (PANC-1, BxPC-3, HPAF-II and SW1990) were obtained from the Cell Bank, Chinese Academy of Sciences (Shanghai, China) and propagated in our laboratory by culturing in complete growth medium as recommended by the supplier. All cell lines were genotyped for identity by Cell Bank, Chinese Academy of Sciences and tested for ruling out mycoplasma contamination. Cells were incubated at 37 °C in atmospheric conditions of 20% O_2_ and 5% CO_2_, treated with 4 μM 5-aza-2′-deoxycitidine (5-Aza-dC) (Sigma, St. Louis, MO, USA) for 3 days, before harvesting for assays for methylation and mRNA expression.

### Animals

All animal experimental protocols were approved by the Ethics Committee for Animal Research of Fuzhou General Hospital of Chinese PLA Nanjing Military Command. (No. FZZY-2016-26). The male athymic nude (BALB/c-nu) mice of 4 to 5 weeks and male NOD/SCID mice of 5 weeks were obtained from Beijing Vital River Laboratory Animal Technology Co., Ltd. (Beijing, China) and housed in a pathogen-free facility of Comparative Medicine Center of Fuzhou General Hospital and maintained on a 12-h light-dark cycle. Food and tap water were provided ad libitum.

### CpG methylation analysis

Genomic DNA was extracted from tissue samples and cell lines with the QIAamp DNA Mini Kit (QIAGEN, Dusseldorf, Germany) according to the manufacturer’s instructions. DNA concentration and purity were determined based on the absorbance at 260 and 280 nm. A total of 1.5 μg of genomic DNA from each sample was converted with sodium bisulfite using an EZ DNA Methylation-Gold Kit (Zymo research, Orange County, CA, USA) according to the manufacturer’s instructions. PCR primers were designed using Epidesigner (http://www.epidesigner.com) and are shown in the Additional file 1: Table S1. Polymerase chain reaction (PCR) was performed with the PCR Accessory Set (Sequenom, San Diego, CA, USA) and the following parameters: hot start at 94 °C for 10 min, followed by denaturing at 94 °C for 45 s, annealing at 62 °C for 48 s, extension at 72 °C for 1 min for 10 cycles, then denaturing at 94 °C for 45 s, annealing at 57 °C for 48 s, extension at 72 °C for 1 min for 35 cycles and final incubation at 72 °C for 3 min. Unincorporated dNTPs were dephosphorylated using the MassCLEAVE Kit (Sequenom). The reaction mixture was incubated at 37 °C for 20 min and shrimp alkaline phosphate (SAP) was then heat inactivated for 5 min at 85 °C. After SAP treatment, 2 μl of the PCR products were used for in vitro transcription and RNase cleavage in accordance with the manufacturer’s instructions (Sequenom). The samples were conditioned and spotted on a 384-pad SpectroCHIP (Sequenom) using the Sequenom MassARRAY platform (CapitalBio, Beijing, China), based on the matrix-assisted laser desorption/ionization time-of-flight (MALDI-TOF) mass spectrometry. Mass spectra were acquired via MassARRAY Compact MALDI-TOF (Sequenom) and the methylation ratios were generated by the EpiTyper software (Sequenom).

### Quantitative real-time PCR (qRT-PCR)

Total RNA was extracted from cells or fresh-frozen tissues with TRIzol reagent (Invitrogen, San Diego, CA, USA) by following the manufacturer’s protocol. The miR-200c and miR-141 levels were quantified by qRT–PCR using TaqMan assay kits (Applied Biosystems, Foster City, CA, USA) with U6 small nuclear RNA as an internal normalization reference. WIPF1, ZEB1, E-Cadherin and Vimentin mRNA was measured by qRT–PCR using a SYBR Premix Ex Taq (Takara, Dalian, China) and normalized for GAPDH expression. The qRT-PCR reactions were performed using an ABI Stepone plus Real-Time PCR System (Applied Biosystems). The primer sequences are listed in the Additional file 1: Table S2.

### Oligonucleotide transfection and sequences of miR-141/200c mimics and anti-miR141/200c mimics

All RNA oligoribonucleotides, including miR-200c mimic, miR-141 mimic, negative control mimic (NC), miR-141/200c inhibitors (anti-miR-141 and anti-miR-200c) and their NC were obtained from Genepharma (Shanghai, China) and their sequences are shown in the Additional file 1: Table S3. The short hairpin RNA (shRNA) sequence for human WIPF1–1 was 5’-GGCCAACAGGGATAATGATTCTTCAAGAGAGAATCATTATCCCTGTTGGCCTT-3′ and WIPF1–2 was GGGAAAGCAGATTCTACTTCCTTCAAGAGAGGAAGTAGAATCTGCTTTCCCTT. Negative Control shRNA vector (Genepharma) was used as a control for RNA interference. The oligonucleotide transfection was performed using the Lipofectamine 2000 reagent (Invitrogen) and transfection was performed according to the manufacturers’ recommendations.

### Lentivirus production and transduction

Human miR-141 mimics, miR-200c mimics and WIPF1 shRNA oligonucleotides were designed and cloned into the LV3-pGLVH1/GFP + Puro vector (GenePharma). The coding sequence of WIPF1 without its 3’-UTR was amplified and cloned into another lentiviral expression vector, LV5-pGCMV/MCS/EF1a/GFP + Puro vector (GenePharma), to produce WIPF1. BxPC-3 and PANC-1 cells were infected with lentiviruses following the instructions of the GenePharma Recombinant Lentivirus Operation Manual. At 72 h after lentivirus infection, the medium was replaced with fresh medium containing puromycin to select for stably infected cells.

### Luciferase reporter assay

The region of the 3′-untranslated region (UTR) of human WIPF1 containing three putative miR-200c binding sites and one putative miR-141 binding site were selected to generate four mutant variants (TargetScan, Additional file 1: Figure S1A and B). Mutant 3’-UTR of human WIPF1 mRNA was generated by site-specific mutagenesis of the wild-type 3’-UTR segment of human WIPF1 mRNA using altered sequence in the complementary site (Additional file 1: Table S1B). The wild-type (WT) and four mutant variants (MUT) derived from the 3’-UTR segment of human WIPF1 mRNA were amplified and subcloned into restriction sites downstream of the luciferase reporter gene in the pmirGlo-vector (GenePharma). Luciferase activities were assayed using a Dual-Luciferase Reporter Assay system (Promega, Madison, WI, USA) according to the manufacturer’s instructions. Transfections were performed in duplicate and repeated at least three times in separate experiments.

### Cell proliferation assay

Cell proliferation was evaluated using the Cell Counting Kit-8 (CCK-8, Dojindo, Tokyo, Japan). BxPC-3 and PANC-1 cells were seeded in 96-well culture plates at 5 × 10^3^ cells/well, transfected with the indicated miRNA and incubated for 1, 2, 3, 4 and 5 days. After washed in PBS, 100 μl medium containing 10% CCK-8 solution was added to each well and incubated for 2 h at 37 °C. Samples are read directly in the wells using an absorbance of the 450 nm wavelength by an enzyme linked immunosorbent assay (ELISA) plate reader.

### Migration and invasion assays

Cell migration and invasion were measured by a Transwell migration plates (24-well, 8 μm pore size, Corning Costar, NY, USA) and chamber invasion assay (Matrigel-coated membrane, Corning Costar). The upper chamber contained pancreatic cancer cells in serum-free medium, and the lower chamber contained culture medium with 10% fetal calf serum. After incubation for 8 h (for migration assay without Matrigel-coated membrane) or 24 h (for invasion assay with Matrigel-coated membrane) at 37 °C, non-invading cells were removed with cotton swabs, and cells that had invaded to the underside of the membrane were stained with 0.1% crystal violet. Then, the invaded cells were counted under an inverted microscope.

### In vivo tumorigenicity assay

Pancreatic cancer cells (1 × 10^7^ cells in 100 μl PBS) were injected subcutaneously into the right axilla of each male athymic nude mice. The length (L), width (W) and height (H) of the tumors were measured weekly. The tumor volume (V) was calculated using the following formula: V = (L × W × H) × 0.5. Mice were sacrificed 28 days after the injection. Tumors were removed and weighed. Each group contained at least five animals.

### Experimental metastasis assay

An in vivo metastasis model was established as previously described [31]. Pancreatic cancer cells (1 × 10^6^ cells in 50 μl PBS) were injected into the spleens of NOD/SCID mice. Ten weeks after the injections, the animals were sacrificed under deep anesthesia (pentobarbital sodium [30 mg·kg^− 1^]) and the liver and lungs were harvested. Tumor metastases were quantified by counting the number of metastatic colonies on one histological section at the middle portion of each liver or lung sample. Each group contained at least five animals.

### Western blot

Western blotting of WIPF1, YAP and TAZ in pancreatic cancer cells was with the methods described previously [32].

### The cancer genome atlas (TCGA) data analysis

RNAseq and clinical data of 177 patients defined by the TCGA pathologist as PDAC were obtained from the TCGA Data Portal (https://tcga-data.nci.nih.gov). All data were downloaded from the November 22, 2017 standard dataset. For RNAseq data, expression levels were TPM-normalized and ENSG-ID transformed.

### Statistical analysis

Quantitative data were expressed as the mean ± standard deviation (SD) and analyzed with variance and Student’s t-test. The relationships between the expression of miRNA and the level of DNA methylation were analyzed with a Spearman correlation coefficient. The Kaplan–Meier method was used to analyze the overall survival (OS) of patients from the TCGA database for correlation with WIPF1 expression, and statistical significance was calculated using the log-rank test. All statistical analyses were performed using SPSS statistical software version 19**.**

## Results

### Quantitative analysis of CpG methylation of miR-200 family in PDAC

**Fig. 1:**
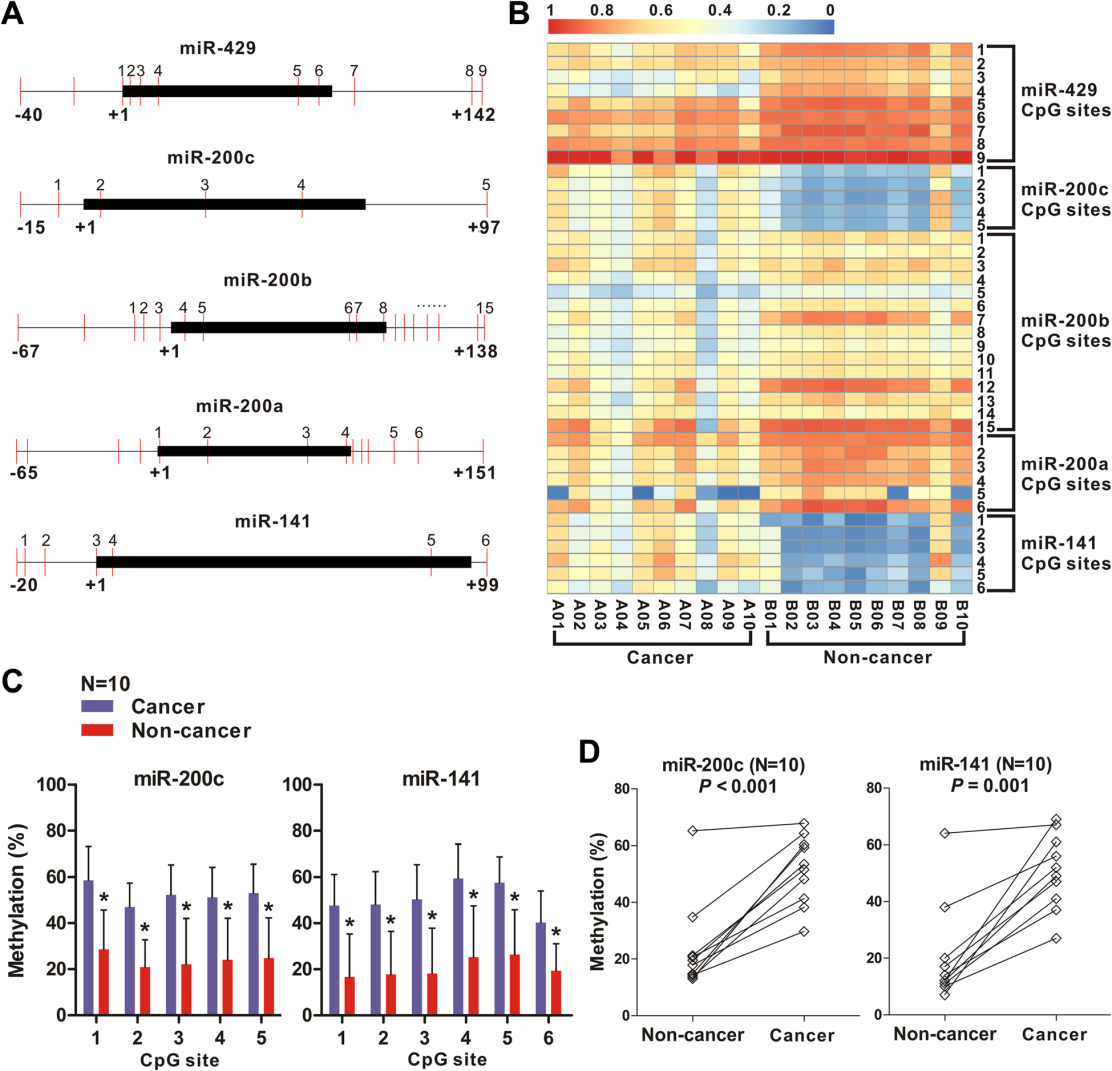
The levels of CpG methylation of the miR-200 family gene promoters in human PDAC. **a** Quantitative analysis of the CpG methylation of the promoters of miR-200 family members (miR-200a, miR-200b, miR-200c, miR-141, and miR-429). The schematic illustration shows the position of the members of the pre-miR-200 (black bar) and their CpG sites. The start site is indicated by + 1. Each red vertical line indicates an individual CpG site. The location of the CpG sites used for the methylation assays are indicated. **b** The levels of CpG methylation of the miR-200 family members in 10 paired PDAC and their surrounding non-cancerous tissues on a heatmap. A01-A10 and B01-B10 represent corresponding10 paired human PDAC (A1-A10) and their surrounding non-cancerous tissues (B1-B10) respectively. The colors displayed represent the relative methylation levels in 0.1 increment, from blue = 0 (0% methylated) to red = 1 (100% methylated). **c** The mean methylation levels of each individual CpG site of miR-200c or miR-141 promoter derived from the 10 paired human PDAC and the surrounding non-cancerous tissues. Error bars are presented as the mean +/− SD (standard deviation). ^*^ *P* < 0.05. **d** The mean methylation levels of the CpG sites of miR-200c or miR-141 promoter of each individual PDAC compared to the paired surrounding non-cancerous tissue. Each circle represents the mean methylation level of the CpG sites of miR-141 or miR-200c. *P* value as indicated

We quantified the promoter DNA methylation levels of the miR-200 family members miR-200a/200b/429 and miR-200c/141 using the MassARRAY compact system with MALDI-TOF mass spectrometry which permits high-throughput identification of methylation sites and semi-quantitative measurement at single or multiple CpG sites. The CpG sites of the promoter region used for the methylation assays in each miR-200 family member are indicated (Fig. 1a). As shown by the heatmap (Fig. 1b), the levels of CpG methylation of miR-200a, miR-200b and miR-429 were significantly lower (hypomethylation) in the 10 paired human pancreatic cancer tissues (A01-A10), compared to the surrounding non-cancerous tissues (B01-B10, hypermethylation). This result is similar to that was previously reported [14]. In contrast, the levels of the CpG methylation of miR-141 and miR-200c were significantly higher in human PDAC. To confirm this result, we derived the mean values from all the CpG sites and found that the levels of CpG methylation of miR-141/200c were significantly higher in the PDAC tissues compared to the surrounding non-cancerous tissues (Fig. 1c). When the mean methylation values were calculated from each individual CpG of all the 10 paired samples, the result was similar (Fig. 1d). The opposite result for the miR-200a/200b/429 promoter was obtained as expected (Additional file 1: Figure S2A and B). Collectively, these results indicated that the miR-141/200c promoter is hypermethylated while which ofmiR-200a/200b/429 is hypomethylated in PDAC.

### Hypermethylation is responsible for the silencing of miR-141 and miR-200c in PDAC

**Fig. 2:**
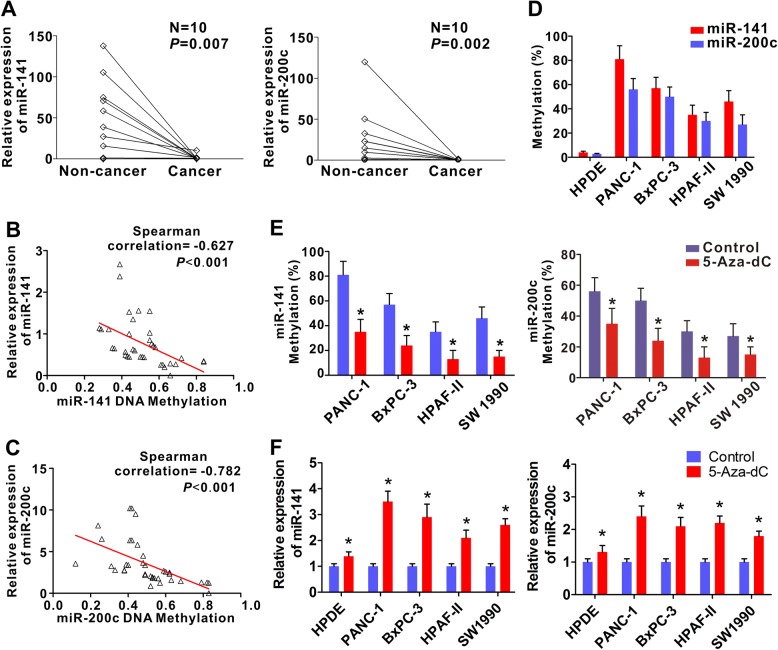
CpG hypermethylation is responsible for the silencing of miR-141 and miR-200c. **a** Expression of miR-141 and miR-200c in 10 paired PDAC and their surrounding non-cancerous tissues. The mRNA expression was measured by qRT–PCR. U6 was used as internal reference. **b** Correlation between the expression of miRNA-141 and its CpG methylation level in 37 PDAC tissues using Spearman’s correlation analysis. **c** Correlation between the expression of miRNA-200c and its CpG methylation level in 37 PDAC tissues using Spearman’s correlation analysis. **d** Levels of methylation of miR-141 and miR-200c promoter region in human pancreatic ductal epithelial cells (HPDE) and pancreatic cancer cell lines (PANC-1, BxPC-3, HPAF-II, and SW1990). Note the levels of methylation in the pancreatic cell lines were all dramatically higher than HPDE cells. The data were derived from three sets of experiments. Error bars are represented as the mean +/− SD. **e** Change of methylation levels of miR-141 and miR-200c promoter in pancreatic cancer cell lines in response to 5-Aza-dC treatment. **f** Relative levels of miR-141 and miR-200c expression in response to 5-Aza-dC treatment in the pancreatic cancer cell lines. For both (**e**) and (**f**), Data were derived from three sets of experiments. Error bars are repreented as the mean +/− SD. ^*^ *P* < 0.05. Control, cells were left untreated; 5-Aza-dC, cells were treated with 5-Aza-dC

To verify whether the CpG hypermethylation mediates the miR-141/200c silencing, we evaluated miR-141 and miR-200c expression in the 10 paired human PDAC tissues by qRT-PCR. As expected, the relative expression levels of miR-141 and miR-200c in PDAC were significantly lower than that of the non-cancerous tissues (Fig. 2a). To further confirm this result, we investigated the correlation between the level of CpG methylation of miR-141 promoter and the level of miR-141 expression in 37 patients with PDAC and found an inverse correlation (*r* = − 0.627, *P* < 0.001, Fig. 2b and Additional file 1: Table S4). Similar result was obtained for miR-200c (*r* = − 0.782, *P* < 0.001, Fig. 2c). We then analyzed the CpG methylation of miR-141 and miR-200c DNA promoter in all the cell lines tested. All four human PDAC cell lines (PANC-1, BxPC-3, HPAF-II and SW1990) showed significantly higher methylation levels, compared to the benign pancreatic tissue human pancreatic duct epithelial cells (HPDE, Fig. 2d). We treated the pancreatic cell lines with the DNA-demethylating agent 5-Aza-dC and found that 5-Aza-dC decreased the level of promoter methylation of miR-141 and miR-200c (Fig. 2e) and restored the expression of miR-141 and miR-200c (Fig. 2f). These results indicated that CpG hypermethylation silenced the expression of miR-141 and miR-200c in PDAC.

### Suppression of PDAC proliferation and metastasis by miR-141 and miR-200c

**Fig. 3:**
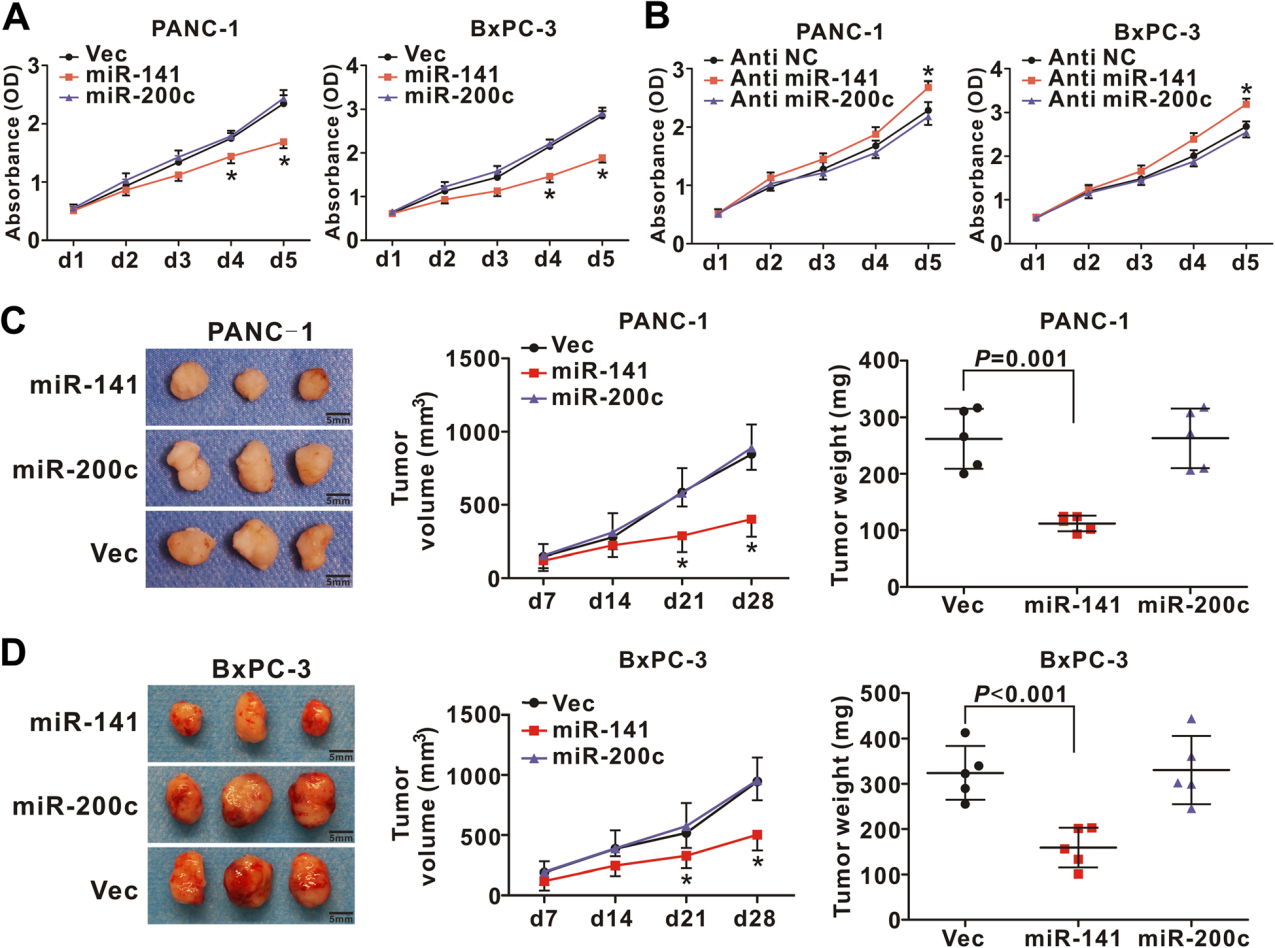
Effect of miR-141 and miR-200c on PDAC cell growth. **a** PANC-1 and BxPC3 cells were stably infected with empty vector as control (Vec) or lentivirus carrying miR-141 or miR-200c and cell proliferation was measured. **b** PANC-1 and BxPC3 cells were stably infected with empty vector or lentivirus carrying anti-miR-141 or anti-miR-200c and cell proliferation was measured. Data represent the mean +/− SD of three independent experiments. ^*^ *P* < 0.05. **c**-**d** miR-141 but not miR-200c suppresses PDAC tumor growth in the xenograft model. PANC-1 (**c**) or BxPC-3 (**d**) cells were infected with empty vector or lentivirus carrying miR-141 or miR-200c and the transfected cells (1 × 10^7^) were injected subcutaneously into the athymic nude mice. Mice were sacrificed 28 days after the injection. Each group contained 5 animals. Tumor volume and weight were measured. ^*^ *P* < 0.05

To determine whether epigenetically regulated miR-141/200c confers tumor-suppressive function in PDAC cells, we stably infected BxPC-3 and PANC-1 cells with a retroviral construct carrying miR-141, miR-200c or with an empty vector as control. We verified the overexpression of these constructs (Additional file 1: Figure S3A and B) and showed that forced expression of anti-miR-141 and anti-miR-200c suppressed the expression of miR-141 and miR-200c (Additional file 1: Figure S3C and D). Using the CCK8 assay, we found that overexpression of miR-141 significantly inhibited cell proliferation while miR-141 inhibitor (anti-miR-141) stimulated cell proliferation (Fig. 3a and b). Interestingly, overexpression of miR-200c or miR-200c inhibitor (anti-miR-200c) showed no significant effect on the cell proliferation (Fig. 3a and b). Using xenograft by injecting the PDAC cells subcutaneously into the athymic nude mice, we found that overexpression of miR-141 but not miR-200c significantly suppressed the tumor growth (Fig. 3c and d).

**Fig. 4:**
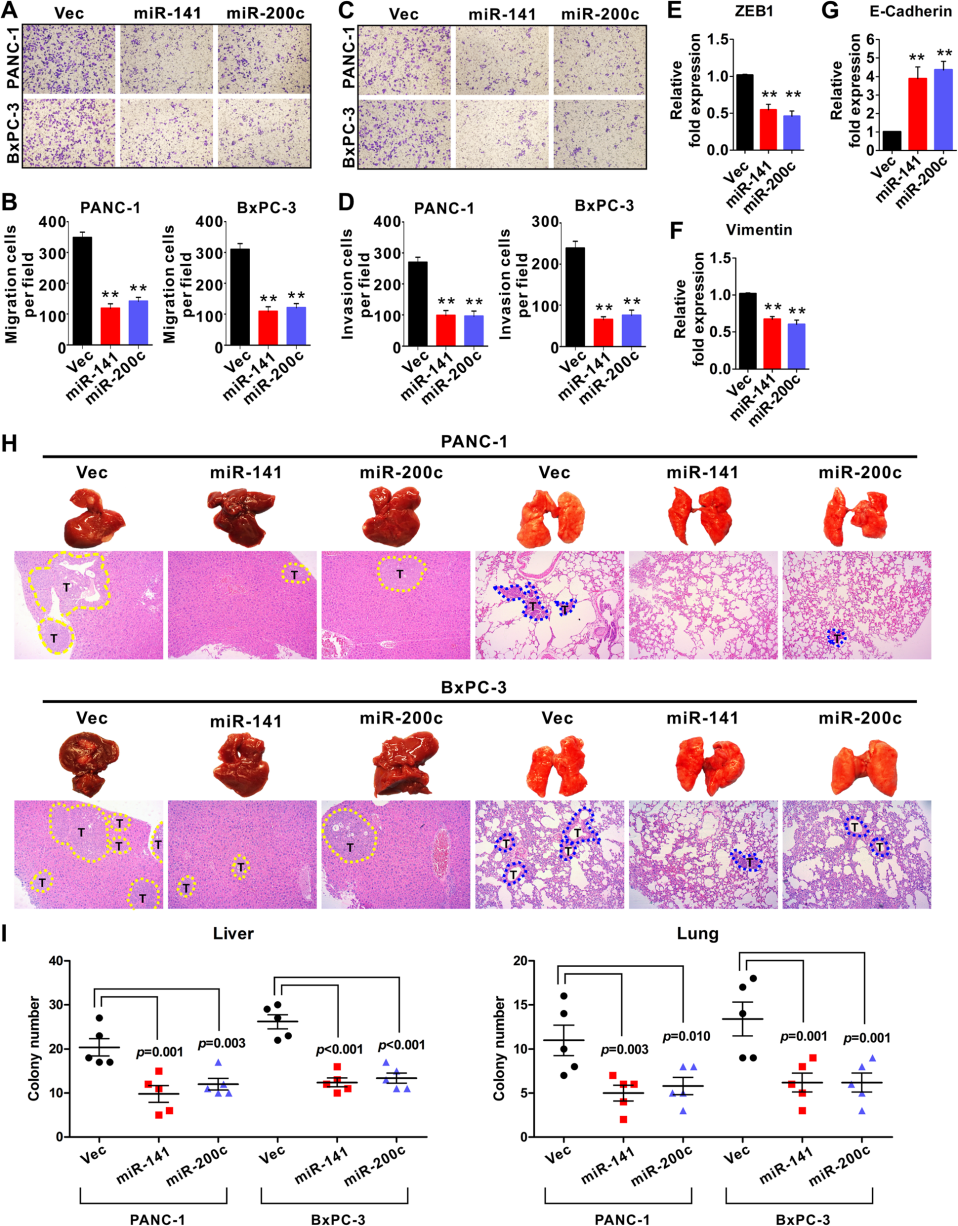
Effect of miR-141 and miR-200c on PDAC cell migration, invasion, and metastasis. **a-b**) miR-141 and miR-200c suppress cell migration; **c-d** miR-141 and miR-200c suppress cell invasion. PANC-1 and BxPC3 cells were used for the assays. The cells were stained and counted in 10 fields. Magnification (× 200). Data represent the mean +/− SD of three independent experiments. ^**^ *P* < 0.01. **e-g** miR-141 and miR-200c affect the expression of EMT markers. PANC-1 cells were treated with miR-141 or miR-200c and RT-PCR was performed to determine the expression of ZEB-1 (**e**), Vimentin (**f**), and E-cadherin (**g**). **h** miR-141 and miR-200c inhibited tumor metastasis in the xenograft model. The experiment was performed with five animals in each group, sacrificed 10 weeks after the injection of cancer cells into the spleen of NOD/SCID mice and liver and lungs were harvested. Representative livers and lungs from mice were shown. H&E staining was performed on sections of metastatic tumors and normal liver or lung tissues. Metastatic tumour nodules (T) outlined by a dotted line. Magnification, × 100. **i** The number of metastatic tumors was quantified by counting the individual tumors on one histological section of the midportion of the liver or lung specimen from each animal. Each group contained five mice

We next investigated the role of miR-141 and miR-200c on the cell migration and invasion of PDAC using transwell chambers with or without Matrigel-coated membranes. Both miR-141 and miR-200c suppressed the migration and invasion of PANC-1 and BxPC-3 cells (Fig. 4a-d). Furthermore, miR-141 inhibitor and miR-200c inhibitor reverse the inhibition (Additional file 1: Figure S4A and B). We also determined that miR-141 and miR-200c suppressed the expression of epithelial-to-mesenchymal transition (EMT) markers ZEB1 and Vimentin while stimulated the expression of E-cadherin, a suppressor of EMT (Fig. 4e-g and Additional file 1: Figure S4C). These results indicate that both miR-141 and miR-200c suppress the stemness of PDAC cells.

To investigate the role of miR-141 and miR-200c on PDAC metastasis, PDAC cells were infected with a lentiviral vector expressing miR-141 or miR-200c, or an empty vector as control and the resulting cells were then injected into the spleens of NOD/SCID mice. Ten weeks after the injection, the animals were euthanized, and the organs were harvested. The overexpressing miR-141 significantly suppressed the tumor growth in the spleen where the cells were injected, while miR-200c had little effect (Additional file 1: Figure S4D). However the mice injected with the cells overexpressing miR-141 or miR-200c had significantly lower number of metastatic nodules in the liver and lungs compared with the mice injected with the cells infected with an empty vector (Fig. 4h and i). These findings demonstrate that miR-141 but not miR-200c retained the ability to inhibit the cell proliferation in vitro and the growth of primary tumor in xenograft, while both were capable of suppressing the migration, invasion and metastasis of PDAC cells.

### WIPF1 is a direct target of miR-141/200c

**Fig. 5:**
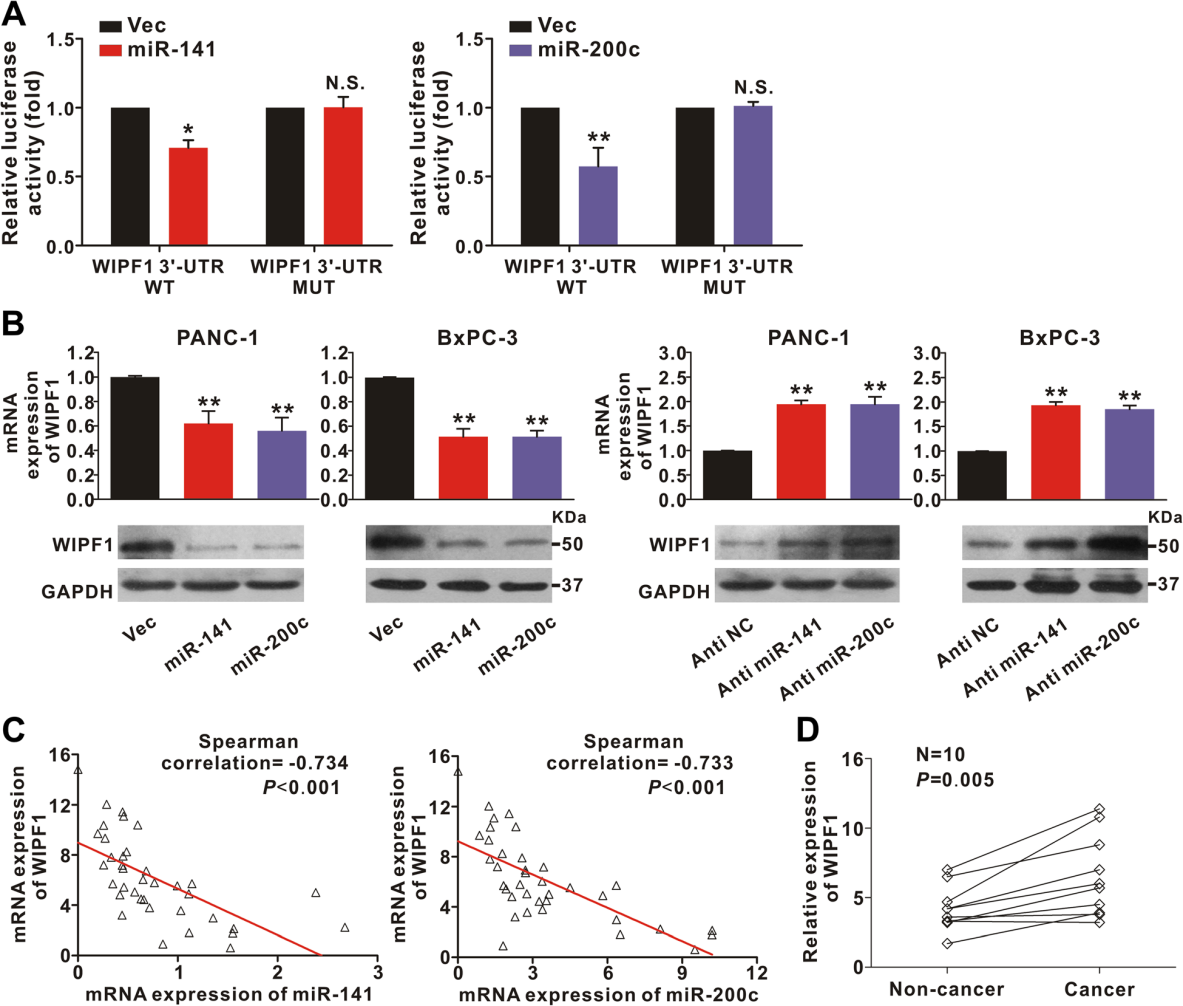
WIPF1 is a direct target of miR-141 and miR-200c. **a** miR-141 and miR-200c inhibit the expression of WIPF1 by targeting its 3-UTR. 293 T cells were transfected with lentivirus miR-141 mimics (left panel) or miR-200c mimics (right panel) and wild type (WT) WIPF1 or mutant WIPF1 with 3’-UTR mutations (MUT), together with luciferase reporter. Luciferase activity was assayed 24 h after transfection. Data was normalized against negative control. ^*^ *P* < 0.05; ^**^ *P* < 0.01; N.S., not significant. **b** miR-141 and miR-200c inhibit the expression of WIPF1 in the pancreatic cell lines. PANC-1 or BxPC3 cells were infected with lentivirus containing miR-141 or miR-200c (left two panels), or anti-miR-141 or anti-miR-200c (right two panels) and harvested for RT-PCR (upper panels) and Western blotting (lower panels). GAPDH was used as normalization control. ^**^ *P* < 0.01. **c** Inverse correlation between the expression of miR-141/200c and the expression of WIPF1 in 37 human pancreatic cancer samples. The expression of miR-141, miR-200c, and WIPF1 were measured by qRT-PCR and analyzed using the Spearman’s correlation analysis. U6 and GAPDH were used as internal normalization references for miR-141/200c and WIPF1 respectively. **d** Expression of WIPF1 in 10 paired PDAC and the surrounding non-cancerous tissues by qRT-PCR. GAPDH was used as an internal normalization reference

To investigate the mechanism underlying the tumor suppressive function of miR-141 and miR-200c, we set out to identify the putative binding sites of their potential target genes. It is well established that microRNAs regulate mRNA expression by targeting the 3′-UTR of mRNAs with a complementary seed sequence [33]. With the use of TargetScan software program, we found that 3′- UTR of WIPF1 contained putative miR-141/200c binding sites (Additional file 1: Figure S1A). To verify their interaction, a dual-luciferase reporter system assay was performed. The wild-type (WT) or the mutant (MUT) miR-141 or miR-200c binding sequences in the 3′-UTR of human WIPF1 (Additional file 1: Figure S1B) was cloned to the downstream of firefly luciferase reporter gene, then co-transfected with miR-141 or miR-200c mimic or an unrelated sequence as control into 293 T cells. As is shown in Fig. 5a, the relative luciferase activity with WT but not the mutant 3′-UTR of WIPF1 was significantly suppressed by the miR-141 or miR-200c mimic. These results indicate that both miR-141 and miR-200c suppress WIPF1 expression through binding to their binding sites on the 3′-UTR of WIPF1. Consistent with these results, using HPDE as positive control we found that both the mRNA and protein levels of WIPF1 in both PANC-1 and BxPC-3 cells were downregulated by the miR-141 and miR-200c mimics, while upregulated by anti-miR-141 and anti-miR-200c (Fig. 5b, and Additional file 1: Figure S5).

To further confirm that WIPF1 is the target of miR-141/200c, we investigated if there is a correlation between their expression. We examined 37 individual human pancreatic cancer tissues and found that the expression of miR-141 inversely correlated with the expression of WIPF1 mRNA (*r* = − 0.734, *p* < 0.001, Fig. 5c, left panel). Similar result was obtained for the expression of miR-200c and WIPF1 (*r* = − 0.733, *p* < 0.001, Fig. 5c, right panel). We also examined 10 paired pancreatic cancer tissues and the surrounding non-cancerous tissues and found that mRNA expression of WIPF1 was highly elevated in the PDAC tissues compared to the surrounding non-cancerous tissues (*P* = 0.005, Fig. 5d). These results indicate that the silencing of miR-141 and miR-200c by CpG methylation in human PDAC leads to increased expression of WIPF1.

### WIPF1 silencing leads to the inhibition of PDAC proliferation, invasion and metastasis

**Fig. 6:**
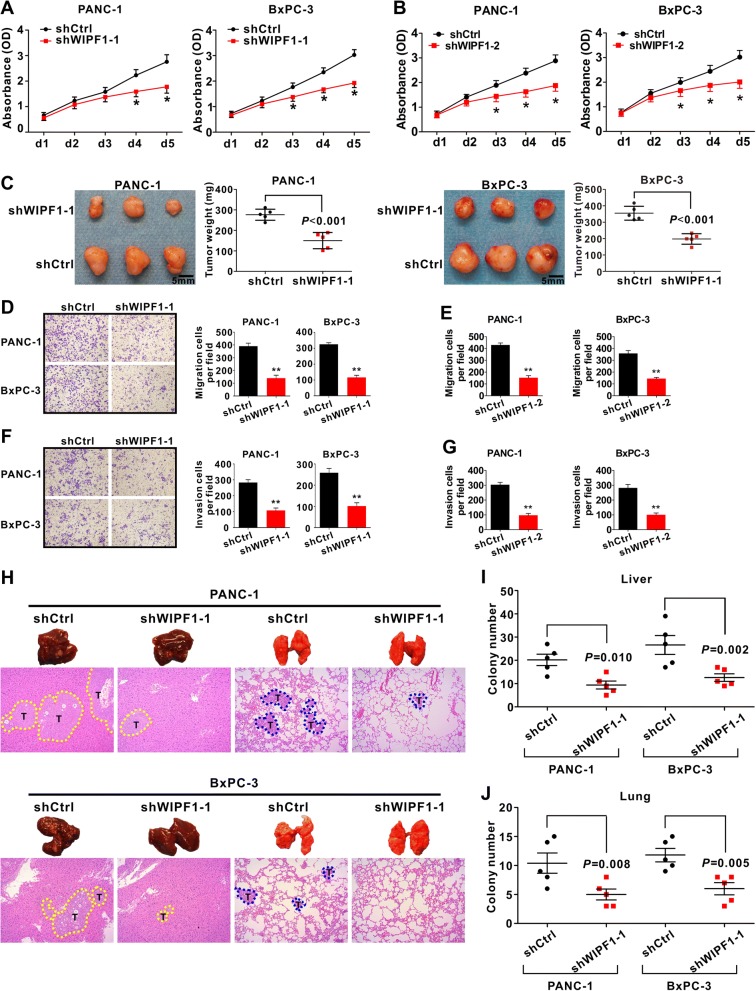
Knockdown of WIPF1 blocks PDAC cell growth and metastasis. **a** and **b** Knockdown of WIPF1 by shWIPF1 inhibits cell growth of PANC-1 and BxPC-3. CCK-8 assay was used to measure cell proliferation. Data represent the mean +/− SD with three independent experiments. ^*^ *P* < 0.05 versus control. **c** Knockdown of WIPF1 inhibits tumor in the xenograft model. Each group contained 5 mice. Tumor weight was measured. **d**-**g** Knockdown of WIPF1 inhibits cell migration (**d** and **e**) and invasion (**f** and **g**) of PANC-1 and BxPC3. Data represent the mean +/− SD with three independent experiments. ^**^ *P* < 0.01. Magnification, × 200. **h**-**j** Knockdown of WIPF1 inhibited tumor metastasis in the xenograft model. Experimental design similar as described in Fig. 4h. Representative livers and lungs from the animals are shown. H&E staining was performed. Metastatic tumour nodules (T) outlined by a dotted line. Magnification, × 100. Student’s t-test was used to analyze the difference between the two groups

To investigate the functional roles of WIPF1 in PDAC, we constructed a cell line with stable knockdown of WIPF1 using a lentivirus vector expressing a short hairpin WIPF1 (shWIPF1) and a control cell line with vector only (shCtrl). Knockdown of WIPF1, as confirmed by the diminished protein and mRNA expression (Additional file 1: Figure S6A-D), reduced cell proliferation (Fig. 6a and b) and tumor growth of primary site (Fig. 6c). This result is nearly identical to that obtained with the overexpression of miR-141 (Fig. 3a-d). Knockdown of WIPF1 also inhibited migration and invasion of both PANC-1 and BxPC-3 cells (Fig. 6d-g), also nearly identical to that obtained with the overexpression of either miR-141 or miR-200c (Fig. 4a-d, h and i). Moreover, knockdown of WIPF1 decreased the number of metastatic nodules in the liver and lungs (Fig. 6h-j). This result is also nearly identical to that obtained with the overexpression of either miR-141 or miR-200c (Fig. 4h and i). These data demonstrate that in opposite to miR-141/200c, WIPF1 promotes cell growth, migration, invasion and metastasis of PDAC cells.

### WIPF1 expression antagonizes the tumor suppressive effect of miR-141/200c and correlates with poor patient survival

**Fig. 7:**
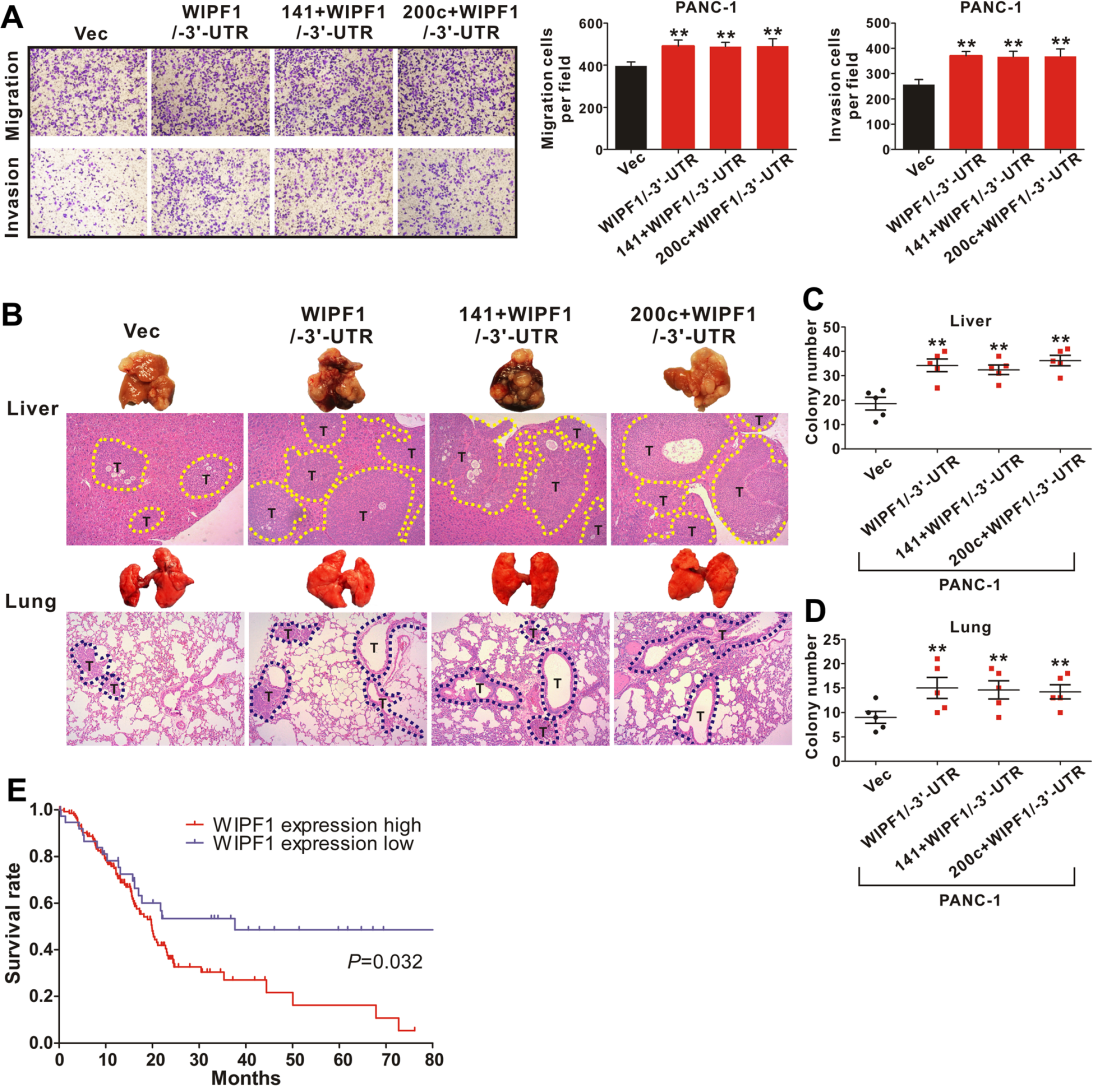
WIPF1 expression antagonizes the tumor suppressive effect of miR-141/200c and correlates with poor patient survival. **a** WIPF1 with the deletion of its 3’-UTR (WIPF1/− 3’-UTR) blocked the inhibitory effect of miR-141/200c on cell migration and invasion of PDAC. PANC-1 cells were infected with either an empty vector as control or lentivirus containing WIPF1 cDNA that encodes the entire coding sequence minus its 3’-UTR, together with lentivirus containing either miR-141 or miR-200c. Data represent the mean ± SD with three independent experiments. ^**^ *P* < 0.01. **b**-**d** Expression of WIPF1/− 3’-UTR blocked the inhibitory effect of miR-141/200c on tumor metastasis. PANC-1 cells were infected as described in (**a**), and the resulting cells were injected into the spleen of NOD/SCID mice. Representative livers and lungs from the animals are shown. H&E staining was performed. Metastatic tumour nodules (T) outlined by a dotted line. Magnification, × 100. Student’s t-test was used to analyze the difference between the two groups. **e** High expression of WIPF1 in PDAC correlates with poor patient survival. Data was derived from 177 patients in the TCGA database. Kaplan-Meier method was used to display the survival curve. Statistical analysis was performed using the log rank test

To determine whether miR-141/200c targets WIPF1 to suppress the invasion and metastasis of PDAC cells, PANC-1 and BxPC-3 cells were transfected with a plasmid carrying the cDNA that encodes the entire coding sequence of WIPF1 but with the deletion of the 3’-UTR (WIPF1/− 3’-UTR). We found that WIPF1/− 3’-UTR was resistant to the suppressive effect of miR-141 and miR-200c on its expression (Additional file 1: Figure S7A). Moreover, WIPF1/− 3’-UTR overcame the suppressive effect of miR-141/200c on the migration, invasion and metastasis of PDAC cells (Fig. 7a-d and Additional file 1: Figure S7B-D). These results demonstrate that WIPF1 is indeed a functional target of miR-141/200c.

To determine the impact of WIPF1 expression on the survival of patients with pancreatic cancer, we identified 177 PDAC cases from the TCGA database and examined the correlation between the level of expression of WIPF1 and overall survival (OS). These patients had a mean age of 64.7 and majority (85%) had stage II and III disease (Additional file 1: Table S5). We found 38 cases with low expression and 139 with high expression of WIPF1 in the tumor tissues (cut-off value = 4.81; the partition was constructed by the length of OS). Using Kaplan-Meier estimate, we found that the patients with low WIPF1 expression was associated with significantly longer OS compared to the patients with high WIPF1 expression (*P* = 0.03). The median OS was 38 months in the low WIPF1 expression group versus 19 months in the high WIPF1 expression group (Fig. 7e). This result indicates that WIPF1 expression is associated with a more aggressive disease course and possibly resistance to treatment in human PDAC.

### WIPF1 mediates the suppression of YAP/TAZ by miR-141/200c

**Fig. 8:**
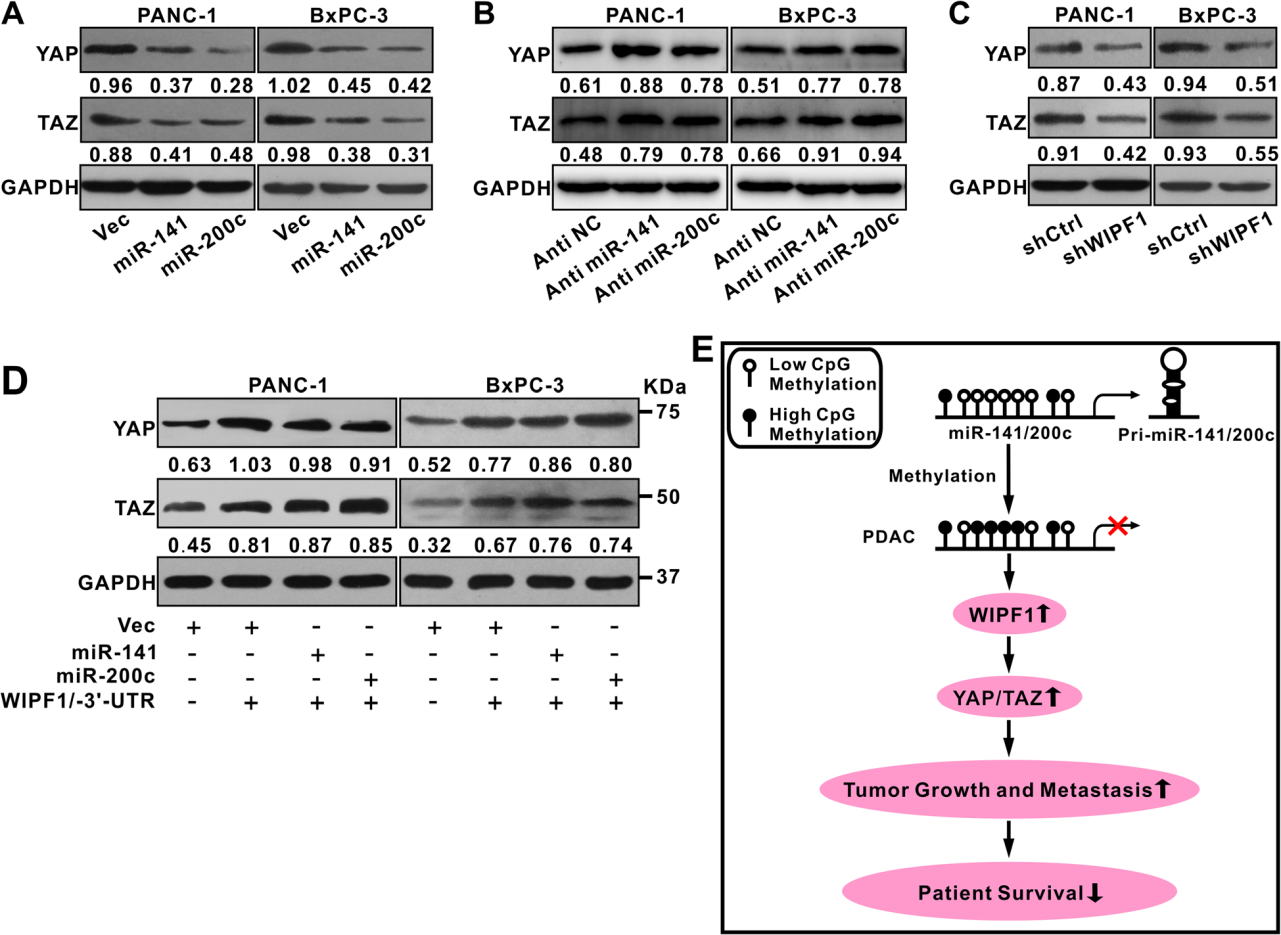
miR-141/200c suppresses YAP/TAZ by repressing WIPF1. **a** miR-141 and miR-200c suppress the expression of YAP/TAZ in PDAC cells. Lentivirus carrying miR-141 or miR-200c was infected into PANC-1 or BxPC-3 cells and the expression of YAP/TAZ was examined by Western Blot. GAPDH was used as reference. **b** Anti-miR-141 and anti-miR-200c increase the expression of YAP/TAZ in PDAC cells. Experimental design similar as described in (**a**). **c** Knockdown of WIPF1 inhibits the expression of YAP/TAZ. PANC-1 or BxPC-3 cells were infected with shCtrl (shControl) or shWIPF1. GAPDH was used as reference. **d** WIPF1 overcomes the suppressive effect of miR-141/miR-200c on YAP/TAZ expression. PANC-1 or BxPC3 cells were infected with empty vector as control or the lentivirus carrying the entire coding sequence of WIPF1 without the 3’-UTR, and with the lentivirus containing miR-141 or miR-200c. GAPDH was used as reference. **e** Schematic illustration demonstrating that the methylation-associated silencing of miR-141/200c in human PDAC promotes tumor growth and metastasis by activating the WIPF1-YAP/TAZ pathway, leading to decreased patient survival

WIPF1 was found to drive tumor progression by stabilizing the YAP/TAZ complex [32]. Previously it was also shown that miR-141 inhibited the expression of YAP1 in pancreatic cancer [17]. We investigated if miR-141/200c suppress YAP/TAZ expression by inhibiting WIPF1. We found that expression of both YAP and TAZ was indeed suppressed by the overexpression of miR-141 or miR-200c in both PANC-1 and BxPC3 cells (Fig. 8a). While miR-141 or miR-200c inhibitor (anti-miR-141 or anti-miR-200c) increased the expression of YAP and TAZ (Fig. 8b). Knockdown of WIPF1 decreased the expression of YAP and TAZ (Fig. 8c). In addition, forced expression of the mutant WIPF1 with the deletion of its 3′-UTR (WIPF1/− 3’-UTR) stimulated the expression of YAP/TAZ and blocked the suppressive effect of miR141/200c on YAP/TAZ expression (Fig. 8d). Hence, WIPF1 mediates the suppressive effect of miR-141/200c on YAP/TAZ. These results demonstrate that silencing of miR-141/200c stimulates PDAC growth and metastasis by activating the WIPF1-YAP/TAZ pathway, leading to poor patient survival (Fig. 8e).

## Discussion

MicroRNA miR-141 and miR-200c are upregulated in several malignancies while downregulated in several others, playing dual regulatory roles on cell growth and differentiation, tumor invasion and metastasis, depending on the cellular context. For example, miR-141/200c is downregulated in triple negative breast cancer and its overexpression stimulates its invasive and migratory property [34]. In non-small cell lung cancer, high expression of miR-141/200c was associated with worse survival [35]. While in renal cell carcinoma, miR-141/200c suppresses cell proliferation and metastasis by targeting EphA2 [36]. In PDAC, miR141 and miR-200c are downregulated and target several genes including YAP1 [17], as well as MAP4K4, MUC1, and TM4SF1 [37]. All these indicate that miR-141 and miR-200c play important roles in regulating cell growth and differentiation as well as metastasis.

We show that the CpG methylation in the promoter region was responsible at least in part for the downregulation of miR-141/200c expression in human PDAC. This was further confirmed by the restoration of miR-141/200c expression by the demethylating agent 5-azacitidine-dC in PDAC cell lines. In contrast, the other cluster of miR-200 family members including miR-200a, miR-200b and miR-429 were hypomethylated in PDAC, suggesting that the different cluster of the same microRNA family may play opposing roles.

We focused our study on the roles of miR-141/200c in PDAC and demonstrated that miR-141 suppressed the cell proliferation and tumor growth of PDAC in in vitro and in xenograft model. Interestingly, our data showed the lack of inhibition on cell proliferation by miR-200c, despite both microRNAs displayed the inhibitory effect on tumor cell invasion, migration, metastasis as well as expression of WIPF1. This suggests that the inhibitory effect on the cell proliferation and the metastasis of PDAC by miR-200c may be two separate and decoupled mechanisms independent of each other and miR-200c is a key regulator for the metastasis but not for primary tumor growth, similar to a previous study [38]. This is consistent with that two transmembrane mucins involved in invasion and metastasis, MUC4 and MUC16, are targeted by miR-200c [18]. A previous study also showed that miR-141 inhibited invasion and migration of PDAC cells but not proliferation [16]. There are likely other unidentified factors that are involved in the miR-200c dependent regulation of primary tumor growth. Importantly, we showed that both miR-141 and miR-200c suppressed the expression of EMT markers while stimulating the expression of tumor suppressor E-cadherin.

Using TargetScan program, we identified WIPF1 as a direct target of miR-141/miR-200c. The 3’-UTR of WIPF1 contains the miR-141 and miR-200c binding sites and the deletion of this 3’-UTR from WIPF1 rendered its expression no longer responsive to the suppression by miR-141/miR-200c. Consistent with this, our data with 37 PDAC cases showed that the expression of miR-141/200c and WIPF1 in human PDAC inversely correlated. WIPF1 binds to the untranslated region of WASP and stabilize its expression [22]. The mutations on the WIPF1 binding site of WASP cause Wiskott-Aldrich syndrome (WAS) with increased susceptibility to leukemia and lymphoma [22]. WIPF1 participates in actin cytoskeleton organization and polymerization that are required for the epithelial-to-mesenchymal transition (EMT) [19–21]. Our data show that silencing of WIPF1 blocks tumor growth and metastasis while high expression of WIPF1 in human PDAC was associated with inferior patient survival, consistent with the previous study that high expression of WIPF1 was associated with poor survival in other types of malignancy [25, 39]. These findings indicate that WIPF1 harbors characteristics of an oncogene and plays important role in promoting tumor growth and metastasis.

Importantly, our study shows that miR-141 and miR200c suppress YAP/TAZ expression by repressing the expression of WIPF1. Forced expression of WIPF1 stimulated the expression of YAP/TAZ and overcame the suppression of YAP/TAZ by miR-141/200c, consistent with a previous study that showed WIPF1 stimulated tumor growth by enhancing YAP/TAZ stability [32]. The YAP/TAZ complex is a key complex of HIPPO pathway and participates in the regulation of other critical signaling transduction pathways such as Wnt/beta-catenin and may serve as a common endpoint of several pathways leading to malignant progression [30, 40–44]. By silencing miR-141/200c, PDAC hijacks a physiologic regulatory checkpoint that is key to preventing deregulated cell growth.

## Conclusion

In conclusion, we have characterized a new oncoprotein expression profile of WIPF1 in PDAC and demonstrated that miR-141/200c regulates the invasion and metastasis of PDAC cells via miR-141/200c-WIPF1-YAP/TAZ pathway. Treatment that combines demethylating agents that are capable of de-repressing miR-141/200c may be a durable strategy for the treatment of metastatic pancreatic cancer. Future studies should also investigate the mechanism of methylation of miR-141/200c.

## Additional file

Additional file 1: **Table S1.** The primer sequences used for polymerase chain reaction. **Table S2.** The nucleotide sequence of the primers used for qRT-PCR. **Table S3.** The sequence of miR-200c mimic, miR-141 mimic, anti-miR-200c mimic (Has-miR-200c inhibitor), and anti-miR-141 mimic (Has-miR-141 inhibitor) used for lentivirus transfection and luciferase reporter assay. **Table S4.** Characteristics of patients with pancreatic cancer (*N* = 37). **Table S5.** Characteristics of patients with pancreatic cancer from the TCGA database (*N* = 177). **Figure S1.** Identifying miR-141/200c target genes using the TargetScan software program. **Figure S2.** The levels of CpG methylation of the promoter region of miR-200a/200b/429 in PDAC. **Figure S3.** Lentiviral expression of miR-141 and miR-200c and their inhibitors in pancreatic cancer cell lines. **Figure S4.** The effect of miR-141 and miR-200c inhibitors on cell migration and invasion in vitro and tumor growth in xenograft. **Figure S5.** miR-141 and miR-200c inhibit the expression of WIPF1 in HPDE cell line. **Figure S6.** Lentiviral expression of shWIPF1 in pancreatic cancer cell lines. **Figure S7.** WIPF1 antagonizes the inhibitory effect of miR-141/200c on cell migration, invasion and metastasis of PDAC. (DOCX 17513 kb)

## Abbreviations

5-Aza-dC: 5-aza-2′-deoxycitidine
Ctrl: Control
EMT: Epithelial-to-mesenchymal transition
HPDE: Human pancreatic duct epithelial cells
miR-200: microRNA-200
MUT: Mutant
NC: Negative control
OS: Overall survival
PDAC: Pancreatic ductal adenocarcinoma
shRNA: Short hairpin RNA
TAZ: Transcriptional coactivator with PDZ-binding motif
TCGA: The cancer genome atlas
UTR: Untranslated region
WAS: Wiskott-Aldrich syndrome
WASP: Wiskott-Aldrich syndrome protein
WIPF1: Wiskott–Aldrich syndrome protein interacting protein family member 1
WIPF1/− 3’-UTR: Entire coding sequence of WIPF1 but with the deletion of the 3’-UTR
WT: Wild-type
YAP: Yes-associated protein
