# Supplementary material for: WIPF1 antagonizes the tumor suppressive effect of miR-141/200c and is associated with poor survival in patients with PDAC
Source: J Exp Clin Cancer Res. 2018 Jul 24;37:167. doi: 10.1186/s13046-018-0848-6 (PMC6056910; doi:10.1186/s13046-018-0848-6)
Supplement: Supplementary file 1 — Table S1. The primer sequences used for polymerase chain reaction. Table S2. The nucleotide sequence of the primers used for qRT-PCR. Table S3. The sequence of miR-200c mimic, miR-141 mimic, anti-miR-200c mimic (Has-miR-200c inhibitor), and anti-miR-141 mimic (Has-miR-141 inhibitor) used for lentivirus transfection and luciferase reporter assay. Table S4. Characteristics of patients with pancreatic cancer (N = 37). Table S5. Characteristics of patients with pancreatic cancer from the TCGA database (N = 177). Figure S1. Identifying miR-141/200c target genes using the TargetScan software program. Figure S2. The levels of CpG methylation of the promoter region of miR-200a/200b/429 in PDAC. Figure S3. Lentiviral expression of miR-141 and miR-200c and their inhibitors in pancreatic cancer cell lines. Figure S4. The effect of miR-141 and miR-200c inhibitors on cell migration and invasion in vitro and tumor growth in xenograft. Figure S5. miR-141 and miR-200c inhibit the expression of WIPF1 in HPDE cell line. Figure S6. Lentiviral expression of shWIPF1 in pancreatic cancer cell lines. Figure S7. WIPF1 antagonizes the inhibitory effect of miR-141/200c on cell migration, invasion and metastasis of PDAC. (DOCX 17513 kb) [file 13046_2018_848_MOESM1_ESM.docx]

**WIPF1 antagonizes the tumor suppressive effect of miR-141/200c and is associated with poor survival in patients with PDAC**

Yu Pan^1#^, Fengchun Lu^1#^, Ping Xiong^2^, Maoen Pan^1^, Zheyang Zhang^3^, Xianchao Lin^1^, Minggui Pan^4^, Heguang Huang^1*^

**Additional file**

**Supplementary Tables S1-S5 and Figures S1-S7**

**Table S1:** The primer sequences used for polymerase chain reaction

|  | **miR-200a-6** | **miR-200b-13** | **miR-200c-13** | **miR-141-17** | **miR-429-5** |
| --- | --- | --- | --- | --- | --- |
| **Left Primer** | GAGGGTGGGTTTGTGTGTAGTT | GGGTGTAGTTGTAGGAGGTTTTGTA | TGTAGTTAGTTAAGGGTTGGGGATT | TATTTAGAGGGGTGAAGGTTAGAGG | GATTAGGGTAGTTGTAGGAGGTGGT |
| **Right Primer** | CAACCCATCCCTAAAATAAAAACTC | CCCCAAAACCCAAAACTAATAACTA | CAACAAAACTCACCAAAAAATATCC | ACCTAATAAATCCAAAACCCACAAT | AAAATAAAACACAAAAACCCCAAAC |
| **Direction** | F | R | R | R | F |
| **LPL** | 22 | 25 | 25 | 25 | 25 |
| **RPL** | 25 | 25 | 25 | 25 | 25 |
| **Target Sequence** | GAGGGTGGGCCTGTGTGCAGTCTCAGGGCCCCCAAGCCCCTCTCCGCCAGCCCTGTCCGGTCCCGGCACCACCCCTGGCTGCTCACCGCTCCGGTTCTTCCCTGGGCTTCCACAGCAGCCCCTGCCTGCCTGGCGGGACCCCACGTCCCTCCCGGGCCCCTGTGAGCATCTTACCGGACAGTGCTGGATTTCCCAGCTTGACTCTAACACTGTCTGGTAACGATGTTCAAAGGTGACCCGCCGCTCGCCGGGGACACCACCGAGGCACATCCGGAGCTCCTACTCCAGGGATGGGCTG | GGGTGCAGCTGCAGGAGGCCTTGCATTCCGGGGTCTCTGAGATGCTGGTGGGGGGCGCCCCAGCCCCCTTCCGAAGGTCACGTCCCCCCGTCACTCTCCCCAGAGCCATCTGGCCCGGACGGGGTGGGGTCCGGTGAGCGGGCTGTGTGGGAGGGGAGTGTGGGGCTCGGCGGGAGGCCCTGGGGGCCGGGACGGGGTCGGCCGGTCGCTGCGTGCAGGGCTCCGCCGTCATCATTACCAGGCAGTATTAGAGACCTGACTCCATCCAATGCTGCCCAGTAAGATGGCCACGGCTGCCCGAGCTGGGTCCGGGGGCCGCCCGGCTGAGGGTTGCATGGGACTCGCTGGGAAGCTCAGTAGCTGGGGGGCCTCGTCCTCCTGGGGCACTGAGGACAGCATCGCCGGAGGGCAGGGGCCCGGGTGAGCCCCACCCGCCAGCCCTGCCGAGTCCAGCAGCCACCAGCTTTGGGTCCTGGGG | TGCAGCCAGCTAAGGGCTGGGGACCTGAGGCGATGGATGTTGCTGACACAGGGACAGGGGCCTCCATCATTACCCGGCAGTATTAGAGACTCCCAACCGCACCCAAACACTGCTGGGTAAGACGAGGGCCCCCGCCCAGCCCGCCCACCTTGGGTCAGGCAGCTTCAGGCCCAGGATCCCTGCGGAAAAGCTGCAGATCCCTGGCTCCCATCCCCCCACCACCCGAGTCCCTGGGGACACTTCCTGGTGAGCCCTGCTG | CATCCAGAGGGGTGAAGGTCAGAGGTTGTTGGTCAGTAGTCCTCCATGGTCTTCAGGGCTCCCTGAAGGTTACTGCCGAGAGAACCCACCCGGGAGCCATCTTTACCAGACAGTGTTAGGAGCTTCACAATTAGACCATCCAACACTGTACTGGAAGATGGACCCAGGGCCGGCCGACAGAGAACTACGGTGCGCGCTCACCAGTTGCTACAGGGGACCCTGGCCACAGGCCAGGTGAGGTCTCTTGAGAGGGGGCGAGCCAACCCCAAGCTCAAGGTCGACTGTGGGTTCTGGATCCACCAGGT | GATCAGGGCAGCTGCAGGAGGTGGCAGGAGCCGCCCCCGGGGCCTTCCCTTCACAGGCCCCGCAGACACCAGCCCAGGACCCGGAGGCCACCCACACCACCGCCGGCCGATGGGCGTCTTACCAGACATGGTTAGACCTGGCCCTCTGTCTAATACTGTCTGGTAAAACCGTCCATCCGCTGCCTGATCACCGTTAGAGGAGAGAGCTGCCTGCCCTGCAGCTCATCAGTGCAAAGCCGCCCGGTCTGGGGCTCTCATGCCCCCCTCACTATGGCCTGTGCCCATCCGCAGCCCATCAGTGCAAAGCCGCCGGGGTCTGGGGCTCTTGTGCCCCACCCT |
| **Taregt Length** | 298 | 478 | 259 | 305 | 339 |
| **Target CpG** | 16 | 31 | 8 | 9 | 16 |
| **CpG Analyzed In T** | 10 | 22 | 7 | 9 | 12 |
| **Left Primer Plus**  **Tag** | aggaagagagGAGGGTGGGTTTGTGTGTAGTT | aggaagagagGGGTGTAGTTGTAGGAGGTTTTGTA | aggaagagagTGTAGTTAGTTAAGGGTTGGGGATT | aggaagagagTATTTAGAGGGGTGAAGGTTAGAGG | aggaagagagGATTAGGGTAGTTGTAGGAGGTGGT |
| **Right Primer Plus**  **Tag** | cagtaatacgactcactatagggagaaggctCAACCCATCCCTAAAATAAAAACTC | cagtaatacgactcactatagggagaaggctCCCCAAAACCCAAAACTAATAACTA | cagtaatacgactcactatagggagaaggctCAACAAAACTCACCAAAAAATATCC | cagtaatacgactcactatagggagaaggctACCTAATAAATCCAAAACCCACAAT | cagtaatacgactcactatagggagaaggctAAAATAAAACACAAAAACCCCAAAC |

| **Table S2:** The nucleotide sequence of the primers used for qRT-PCR |
| --- |

| Target Gene |  | Oligonucleotide Primers (5'-3') | Length |
| --- | --- | --- | --- |
| WIPF1 | H-WIPF1-FO-1 | AATGGTGCCTTACTTTGTGATT | 223 |
|  | H-WIPF1-RE-1 | TTTCTTCCTCTACGGTCCTTG |  |
| ZEB1 | ZEB1-FO | GGAGGATGACACAGGAAAGGAA | 180 |
|  | ZEB1-RE | GCCTCAGGAAAAATGACAGCAG |  |
| E-Cadherin | E-Cadherin-FO | TTTGACGCCGAGAGCTACAC | 217 |
|  | E-Cadherin-RE | CCCAGGCGTAGACCAAGAAA |  |
| Vimentin | Vimentin-FO | GGACCAGCTAACCAACGACA | 178 |
|  | Vimentin-RE | AAGGTCAAGACGTGCCAGAG |  |
| HGAPDH | HGAPDH-FO | CATGAGAAGTATGACAACAGCCT | 113 |
|  | HGAPDH-RE | AGTCCTTCCACGATACCAAAGT |  |
| miR-200c | HmiR-200c-FO-2 | GATCGTCA+TAA+TACTGCCG | 81 |
|  | HmiR-RE-4 | ATGGAGCCTGGGACGAGA |  |
| miR-141 | HmiR-141-FO | AGCCGCTAACACTGTCTGGTA | 79 |
|  | HmiR-RE-6 | ATGGAGCCTGGGACGAGAC |  |
| U6 | H-U6-FO | ATTGGAACGATACAGAGAAGATT | 80 |
|  | H-U6-RE | GGAACGCTTCACGAATTTG |  |

**Table S3.** The sequence of miR-200c mimic, miR-141 mimic, anti-miR-200c mimic (Has-miR-200c inhibitor), and anti-miR-141 mimic (Has-miR-141 inhibitor) used for lentivirus transfection and luciferase reporter assay.

**Gene** **Primers (5'-3')**

Has-miR-200c mimic FOR UAAUACUGCCGGGUAAUGAUGGA

REV CAUCAUUACCCGGCAGUAUUAUU

Has-miR-141 mimic FOR UAACACUGUCUGGUAAAGAUGG

REV AUCUUUACCAGACAGUGUUAUU

Has-miR-200c inhibitor FOR UAACACUGUCUGGUAAAGAUGG

REV CCAUCUUUACCAGACAGUGUUA

Has-miR-141 inhibitor FOR UAAUACUGCCGGGUAAUGAUGGA

REV UCCAUCAUUACCCGGCAGUAUUA

_____________________________________________________________________

| **Table S4:** Characteristics of patients  with pancreatic cancer (N=37) | |
| --- | --- |
| Age at enrollment (y) | |
| Mean (SD) | 63.9 (8.4) |
| Range | 50-79 |
| Gender, no. (%) | |
| Male | 21 (56.8%) |
| Female | 16 (43.2%) |
| TNM stage, no. (%) | |
| I | 2 (5.4%) |
| IIA | 5 (13.5%) |
| IIB | 20 (54.1%) |
| III | 10 (27.0%) |
| Grading, no. (%) |  |
| G1 | 1 (2.7%) |
| G2 | 25(67.6%) |
| G3 | 11(29.7%) |

| **Table S5:** Characteristics of patients with pancreatic cancer from the TCGA database (N=177)  ___________________________  Age at enrollment (y) | |
| --- | --- |
| Mean (SD) | 64.7 (10.8) |
| Range | 35-88 |
| Gender, no. (%) | |
| Male | 97 (54.8%) |
| Female | 80 (45.2%) |
| TNM stage, no. (%) | |
| I | 22 (12.4%) |
| IIA | 29 (16.4%) |
| IIB | 119 (67.2%) |
| III | 3 (1.7%) |
| IV | 4 (2.3%) |
| Grading, no. (%) |  |
| G1 | 31 (17.5%) |
| G2 | 95 (53.7%) |
| G3 | 49 (27.7%) |
| G4 | 2 (1.1%) |

**
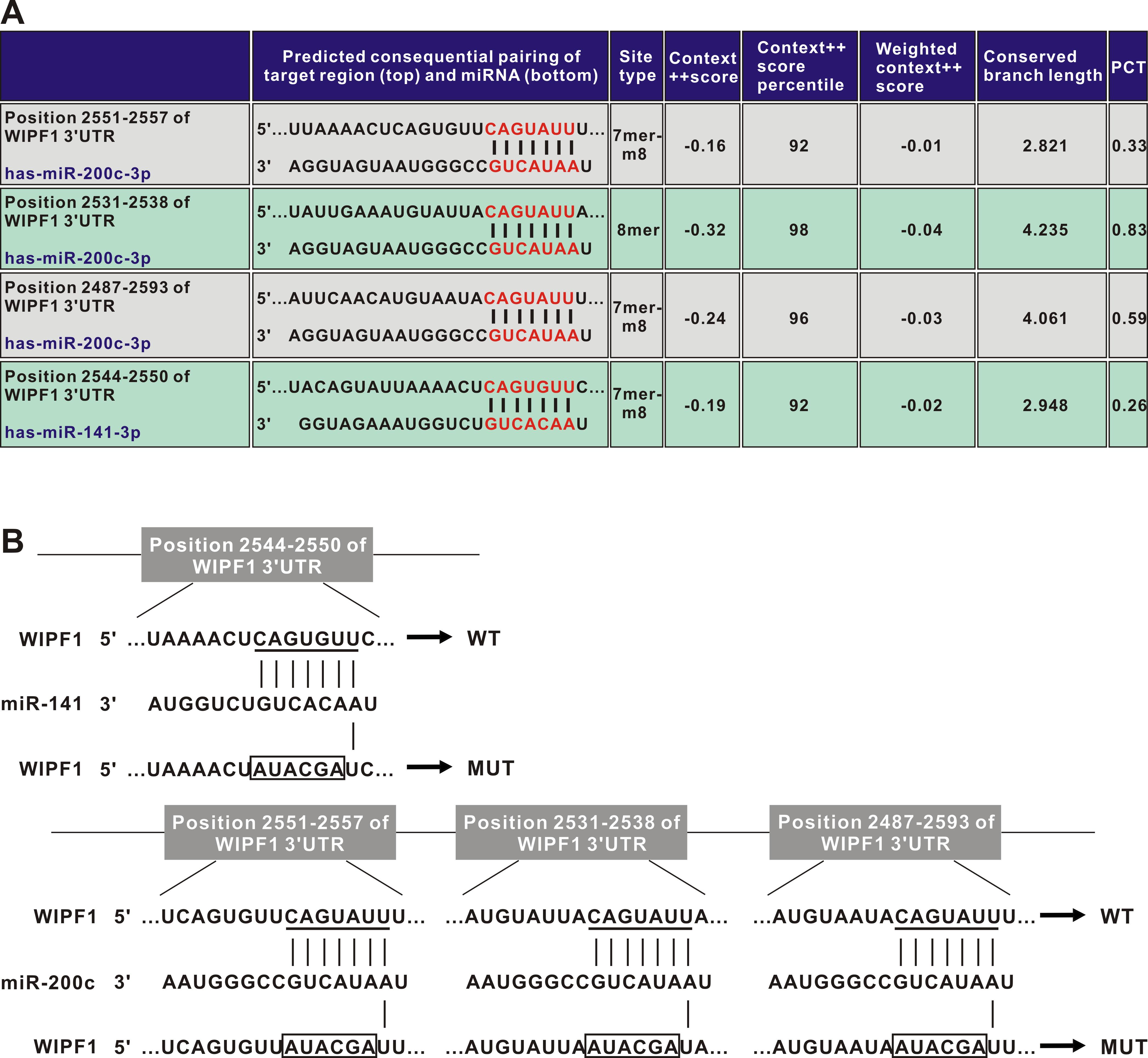
**

**Figure S1.** **Identifying miR-141/200c target genes using the TargetScan software program.** (**A**) The 3’-untranslated region (3’-UTR) of human WIPF1 contains one putative miR-141-binding site and three putative miR-200c-binding sites. (**B**) The sequences of wild-type and mutant 3’-UTR of human WIPF1 that were cloned downstream to the luciferase reporter gene. The position and nucleotide sequences of wild-type and mutant 3’-UTR of WIPF1 in the miR-141 or miR-200c binding site are indicated.


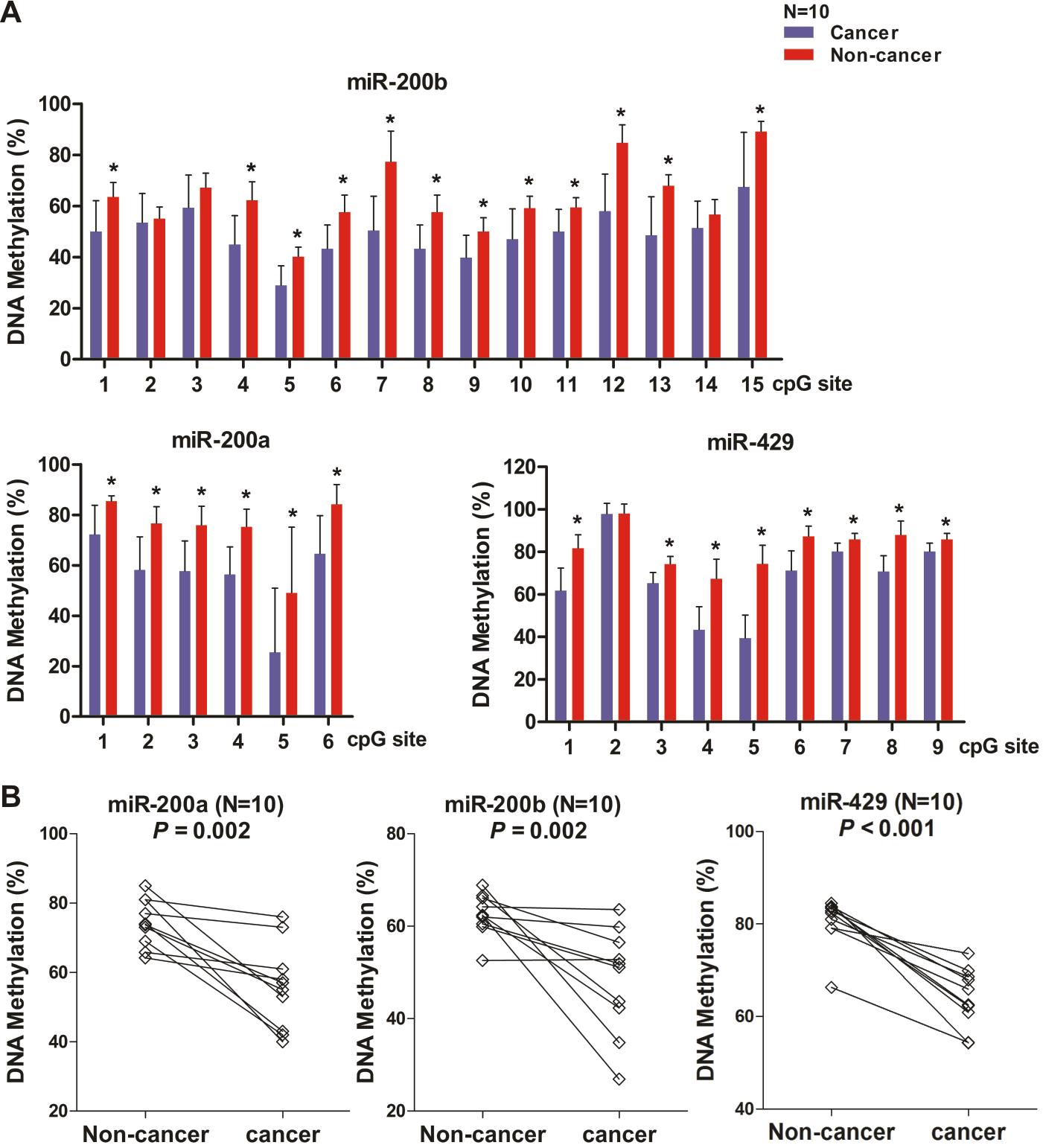


**Figure S2.** **The levels of CpG methylation of the promoter region of miR-200a/200b/429 in PDAC.**

(**A**) The average levels of CpG methylation of miR-200a/200b/429 of 10 paired human PDAC and the surrounding non-cancerous tissues. (**B**) The mean levels of methylation of all the CpG sites of miR-200a/200b/429 in 10 paired PDAC tissues and the surrounding non-cancerous tissues.


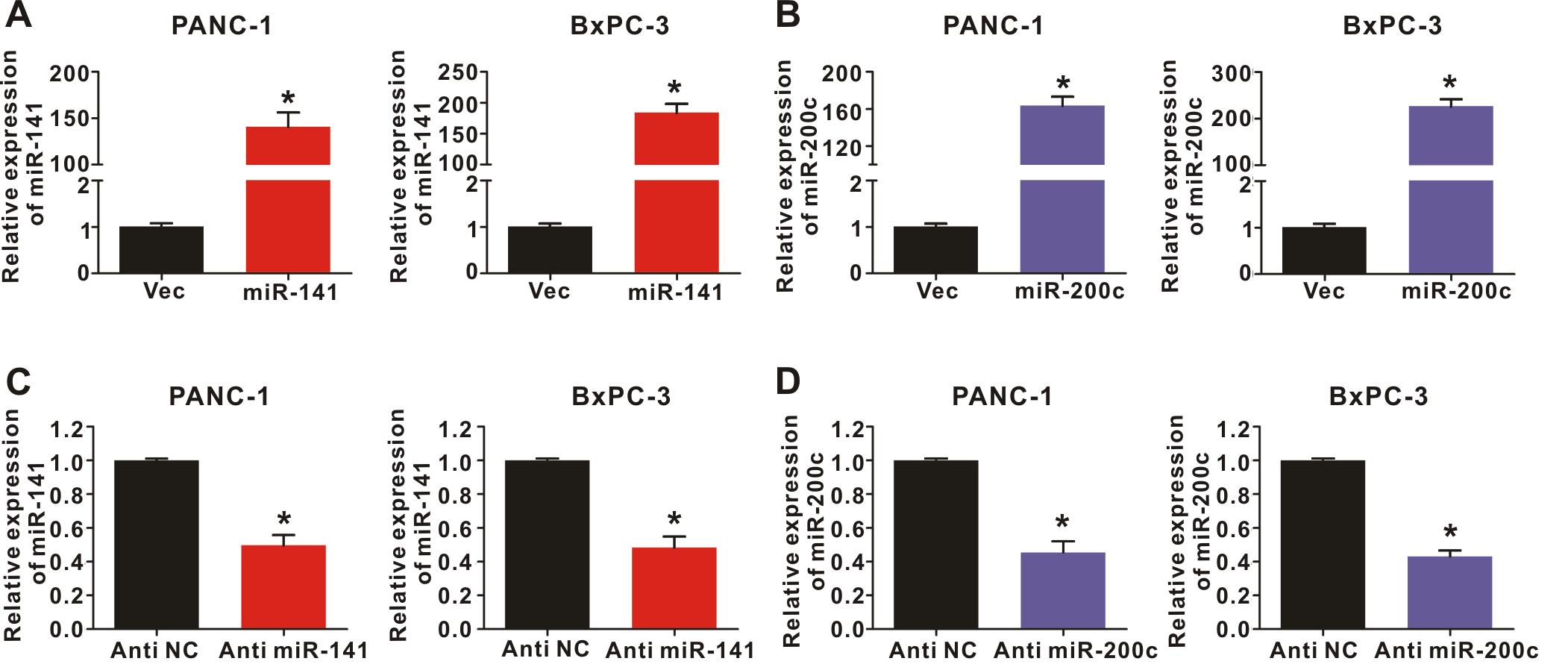


**Figure S3. Lentiviral expression of miR-141 and miR-200c and their inhibitors in pancreatic cancer cell lines.**

(**A**-**B**) Expression of miR-141 (**A**) and miR-200c (**B**) in PANC-1 and BxPC3 cells stably infected with miR-141 or miR-200c lentivirus construct. (**C**-**D**) PANC-1 and BxPC-3 cells were transiently infected with an anti miR-141 inhibitor (**C**) or anti-miR-200c inhibitor (**D**), or negative control (Anti-NC) using Lipofectamine. The levels were measured by qRT-PCR using U6 as an internal normalization reference and empty lentivirus vector as control.


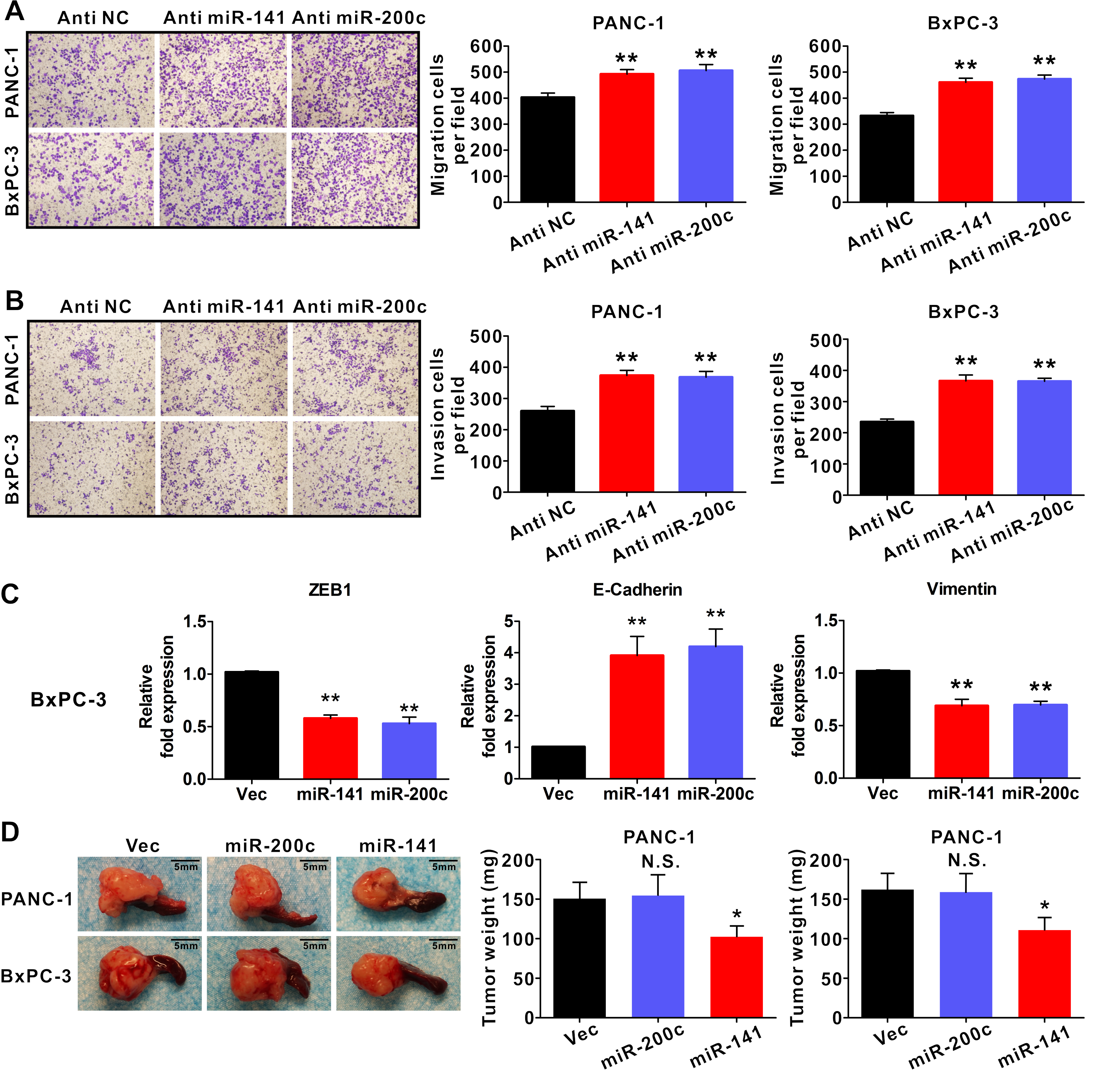


**Figure S4**. **The effect of miR-141 and miR-200c inhibitors on cell migration and invasion *in vitro* and tumor growth in xenograft.**

(**A**-**B**) PANC-1 and BxPC-3 cells were infected with anti-miR-141 or anti-miR-200c. Migration (**A**) and invasion (**B**) assays were performed as described in the Method section. Data represent the mean +/- SD of three independent experiments. ^**^ *P* < 0.01 versus control. Magnifciation, × 200. (**C**) BxPC-3 cells were treated with miR-141 or miR-200c and RT-PCR was performed to determine the expression of ZEB-1, E-cadherin, and Vimentin. (**D**) The effects of miR-141 and miR-200c on spleen-implanted tumor growth. Tumor cells were infected with lentivisus carrying miR-141 or miR-200c and the resulting cells were injected into the spleens of NOD/SCID mice. Ten weeks after the injection, the animals were euthanized and the primary tumors were harvested. The differences in the mean weights of the primary tumors between groups were compared using Student’s t-test. ^*^ *P* < 0.05, versus control. N.S., not significant.


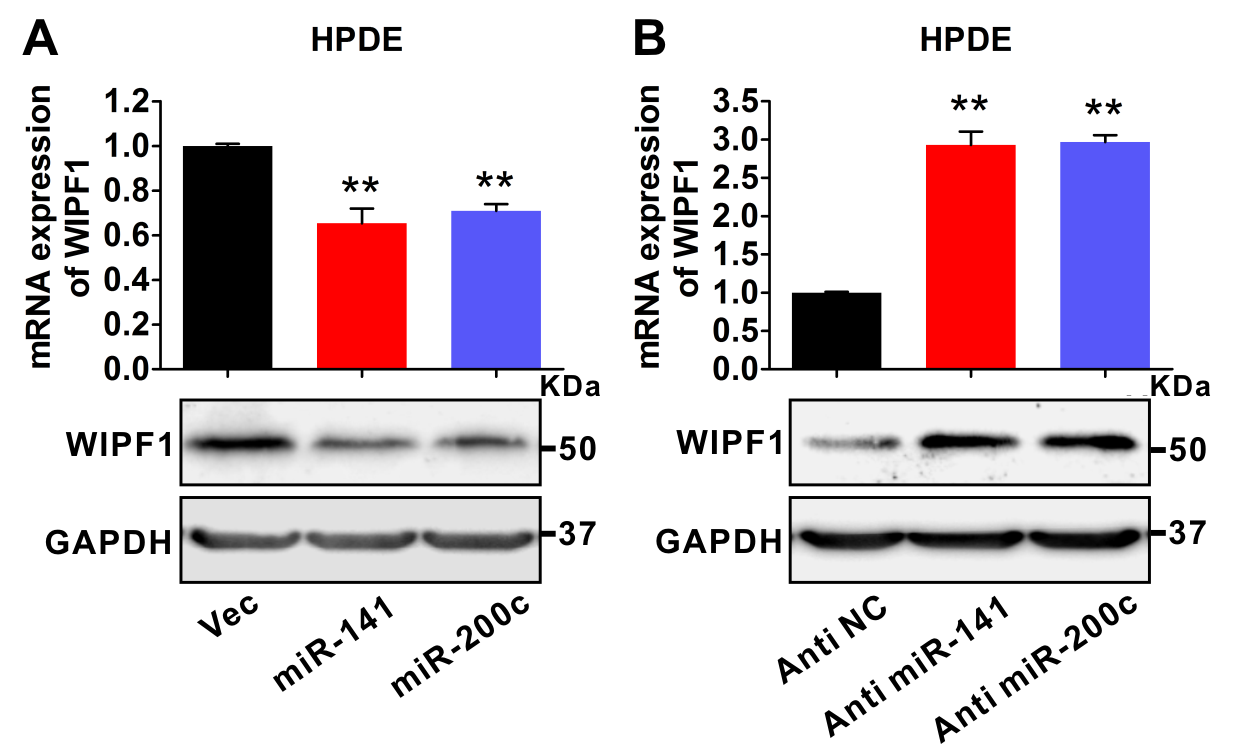


**Figure S5. miR-141 and miR-200c inhibit the expression of WIPF1 in HPDE cell line.** (**A-B**) HPDE cell was infected with lentivirus containing miR-141 or miR-200c (**A**), or anti-miR-141 or anti-miR-200c (**B**) and harvested for RT-PCR (upper panels) and Western blotting (lower panels). GAPDH was used as normalization control. ^**^ *P* < 0.01.


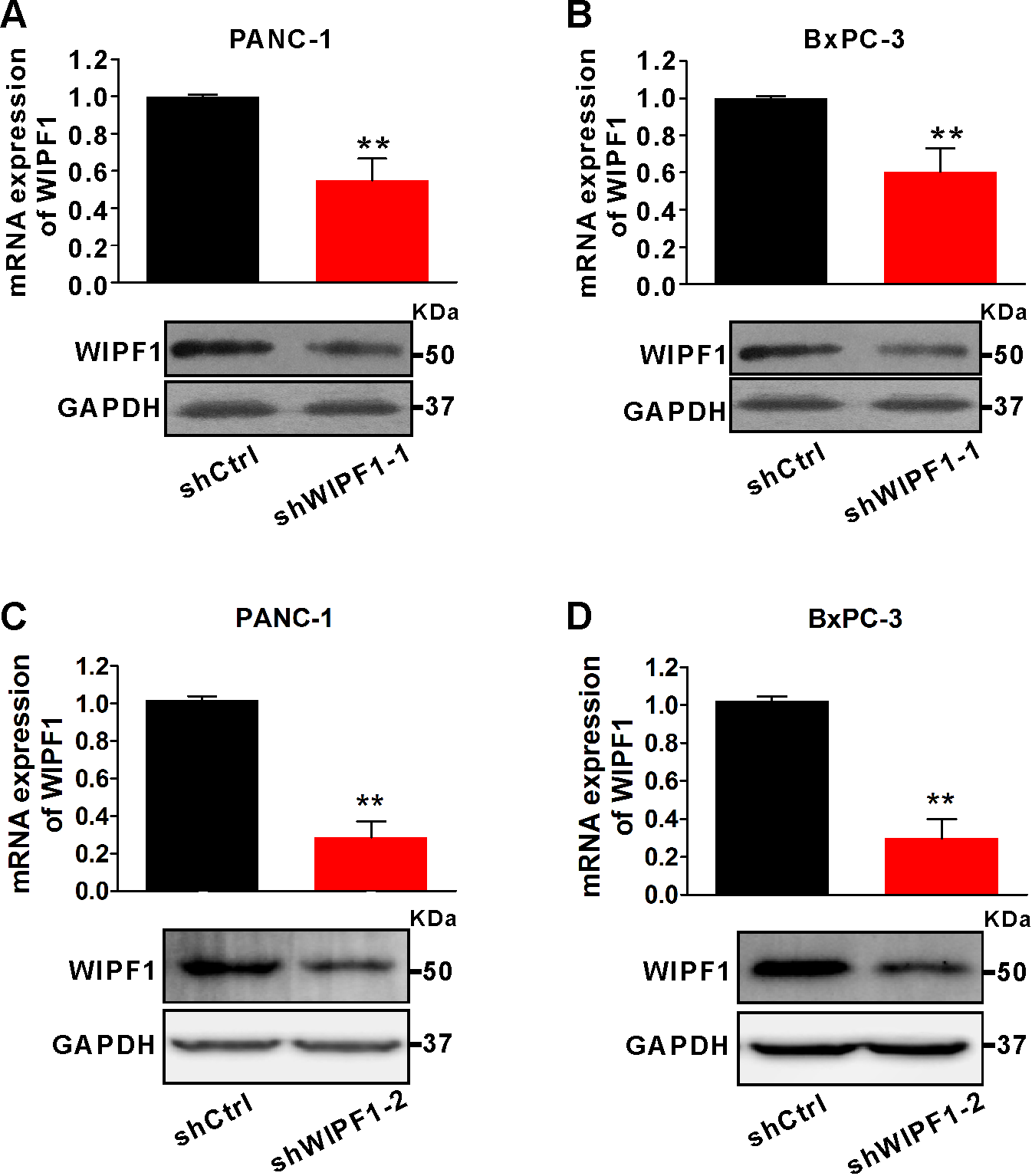


**Figure S6. Lentiviral expression of shWIPF1** **in pancreatic cancer cell lines.**

(**A-D**) Knockdown of WIPF1 by shWIPF1-1 and shWIPF1-2 in PANC-1 (**A** and **C**) and BxPC-3 (**B** and **D**) cells. Cells were infected with lentivirus containing shCtrl or shWIPF1. WIPF1 expression was measured by qRT–PCR (upper panels) and Western blot (lower panels). GAPDH was used as an intern normalization reference. ^**^ *P* < 0.01 versus negative control (shCtrl).


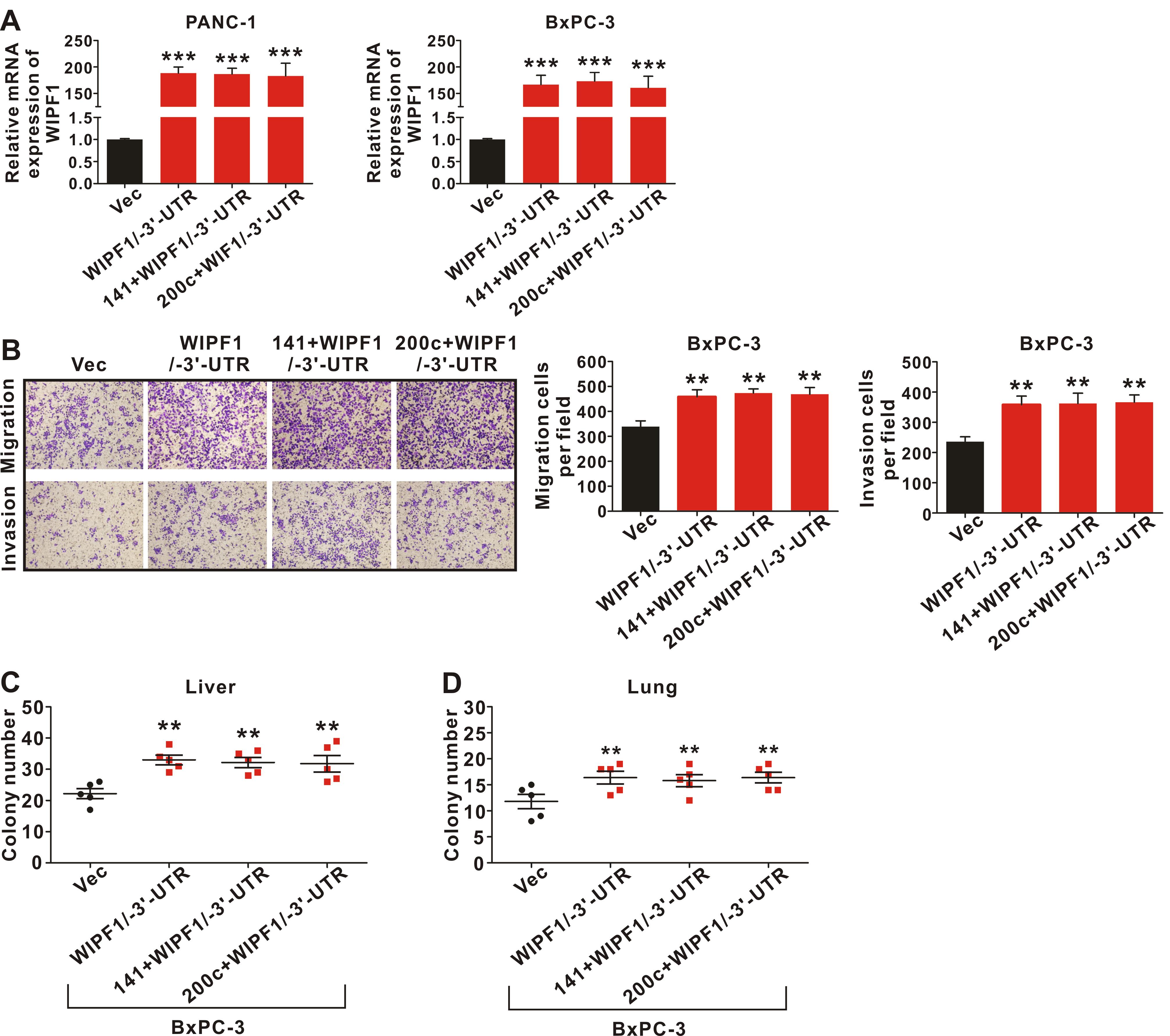


**Figure S7. WIPF1 antagonizes the inhibitory effect of miR-141/200c on cell migration, invasion and metastasis of PDAC.** (**A**) WIPF1 without 3-UTR ameliorates the inhibitory effect of miR-141/200c on its expression in PDAC cells. WIPF1 expression was measured by qRT-PCR. (**B**) BxPC-3 cells infected with vector alone, or with WIPF1, or with WIPF1 plus miR-141 or miR-200c (as described in Fig. 7A). Migration and invasion assays were performed as previously described. Magnification, ×200. Data represent the mean ± SD of three independent experiments. ^**^ *P* < 0.01 versus vector only (Vec). (**C**-**D**) BxPC-3 cells infected with vector alone, or with WIPF1, or with WIPF1 plus miR-141 or miR-200c, and the resulting cells were injected into the spleen of NOD/SCID mice. Organs were harvested 10 weeks after the injection. Each group contained 5 animals. Student’s t-test was used to analyze the statistical differences.
